# Exosomal Wnt-induced dedifferentiation of colorectal cancer cells contributes to chemotherapy resistance

**DOI:** 10.1038/s41388-018-0557-9

**Published:** 2018-11-02

**Authors:** Y.-B. Hu, C. Yan, L. Mu, Y.–L. Mi, H. Zhao, H. Hu, X.-L. Li, D.-D. Tao, Y.-Q. Wu, J.-P. Gong, J.-C. Qin

**Affiliations:** 1 Department of Surgery, Tongji Hospital, Tongji Medical College, Huazhong University of Science and Technology, Wuhan, 430030 China; 2 Molecular Medicine Center, Tongji Hospital, Tongji Medical College, Huazhong University of Science and Technology, Wuhan, 430030 China

**Keywords:** Cancer stem cells, Cancer therapeutic resistance, Cell biology, Cancer microenvironment

## Abstract

Cancer stem cells (CSCs) are inherently resistant to chemotherapy, and CSCs in chemotherapy-failed recurrent tumors are enriched; however, the cellular origin of chemotherapy-induced CSC enrichment remains unclear. Communication with stromal fibroblasts may induce cancer cell dedifferentiation into CSCs through secreted factors. We recently demonstrated that fibroblast-derived exosomes promote chemoresistance in colorectal cancer (CRC). Here, we report that fibroblasts confer CRC chemoresistance via exosome-induced reprogramming (dedifferentiation) of bulk CRC cells to phenotypic and functional CSCs. At the molecular level, we provided evidence that the major reprogramming regulators in fibroblast-exosomes are Wnts. Exosomal Wnts were found to increase Wnt activity and drug resistance in differentiated CRC cells, and inhibiting Wnt release diminished this effect in vitro and in vivo. Together, our results indicate that exosomal Wnts derived from fibroblasts could induce the dedifferentiation of cancer cells to promote chemoresistance in CRC, and suggest that interfering with exosomal Wnt signaling may help to improve chemosensitivity and the therapeutic window.

## Introduction

Colorectal cancer (CRC) is one of the leading causes of cancer-related deaths worldwide, and the mortality rate has been steadily increasing over the past decades [1]. Chemotherapy and targeted therapies have not dramatically improved the clinical outcomes of patients with recurrent or metastatic CRC. A better understanding of the mechanisms underlying chemotherapy resistance in CRC is imperative for the development of more effective therapeutic approaches to benefit CRC patients.

CRC is heterogeneous, manifesting variegated cellular morphologies and histopathological presentations. Experimental evidence for cancer stem cells (CSCs) in CRC was provided using primary human CRC tumor samples [2, 3]. CSCs are hypothesized to be inherently resistant to chemotherapy. Recurrent CRCs after chemotherapy failure are frequently enriched for cells expressing CSC markers [4, 5]. Nevertheless, the cell-of-origin from which increased CSCs are derived in recurrent CRCs remains elusive. The tumor microenvironment is intimately involved in tumor maintenance and progression. Cancer-associated fibroblasts (CAFs), one of the main stroma cells, may induce the dedifferentiation of differentiated CRC cells into CSCs [6]. In addition, CAFs have been shown to promote resistance to chemotherapeutic agents in cancer patients through the secretion of paracrine factors [7]. We recently demonstrated that fibroblast-derived exosomes promote chemoresistance in colorectal cancers (CRCs) [8], but the signaling mechanisms underlying this interesting phenomenon remain unclear.

Exosomes are small membrane vesicles secreted from many different types of cells; they range in diameter from 30 to 100 nm [9]. In addition to being soluble mediators of cell–cell communication, secreted exosomes transport bioactive molecules, such as microRNAs, mRNAs and proteins, to recipient cells [9]. In CRC, exosomes released by CAFs play an important role in cancer progression, including chemoresistance, by transferring their secretions to cancer cells [10]. Although exosomal nucleic acids are well documented [11], the role of proteins in exosomes is less clear. Wnts are a group of evolutionarily conserved glycoproteins that play important roles in stromal and epithelial crosstalk [12]. Wnts are lipid modified and highly hydrophobic, limiting their extracellular dispersal ability [13]. Recent studies have shown that exosomes are capable of transferring insoluble Wnts between diverse cell types [14]. Wnts activate the Wnt/β-catenin pathway by promoting the stabilization and nuclear translocation of β-catenin, which, together with TCF/LEF, activates Wnt target genes [6, 15]. Wnt signaling activity is a functional marker for CSCs and is necessary for maintaining stemness in colon cancer [6]. However, the role of exosomal Wnts in conferring the CSC phenotype remains unclear. Thus, we hypothesized that CAF-derived exosomes may contribute to the secretion of Wnt ligands and thereby regulate Wnt activity to influence treatment resistance in colon cancer.

In the present study, we first examined whether CAFs promoted tumor growth in phenotypically differentiated CRC cells via paracrine signaling during chemotherapy. Dedifferentiation was validated in vitro and in vivo. Exosomes derived from CAFs were found to be required for this paracrine signaling. We then investigated the expression of Wnts in CAFs and their exosomes, and whether exogenous Wnt ligands enhanced the self-renewal ability of the differentiated CRC cells. Wnt-absent exosomes lost the ability to increase Wnt activity in differentiated CRC cells. In vivo studies provided direct evidence that exosomal Wnts played a key role in chemoresistance of differentiated CRC cells.

## Results

### Fibroblasts promote drug resistance in CRC cells via paracrine signaling

To examine the role of the tumor microenvironment in CRC chemoresistance, we subcutaneously (SC) implanted HT-29 CRC cells [16] with or without 18Co cells, a non-tumorigenic colonic fibroblast cell line [6] pretreated with mitomycin C (MMC) to interrupt cell propagation, into female nude mice to model stroma-mediated resistance (Supplementary Figure S1a). All mice were treated with 5-Fluorouracil (5-Fu) to maintain chemotherapeutic stress and resistance in CRC tumors. Xenografts of HT-29 cells co-implanted with MMC-pretreated 18Co cells grew faster and generated larger tumors than those from HT-29 cells alone (Supplementary Figure S1b). To determine whether the tumor-promoting effects of 18Co fibroblasts may have been mediated by direct cell–cell contact and/or paracrine pathways, we made conditioned medium from untreated (CM) or 5-Fu-treated (CM (5-Fu)) 18Co cells (Supplementary Figure S1c), and fresh medium cultured without 18Co cell as the control medium. And we observed that two types of CM both increased the efficiency of sphere formation in HT-29 cells during chemotherapy compared with the control medium (Supplementary Figure S1d). To further determine whether these observations were of relevance to tumor therapy in vivo, we treated HT-29 tumor-bearing mice with 5-Fu together with 18Co-CM or control medium. Administering 18Co-CM strongly promoted the growth of 5-Fu-resistant tumors compared with cells in the control medium (Supplementary Figure S1e).

To extend these observations to a clinical context, we first separated and cultured primary CAFs from CRC patient tumors, and further confirmed that the CAFs were positive for fibroblast makers such as vimentin, FAP-1, and α-SMA, and negative for several epithelial cell markers including EpCAM, CDX2, and CK7 (Supplementary Figure S1f). As shown in Supplementary Figures S1g and h, CAFs-derived CM promoted sphere formation upon administration of 5-Fu or oxaliplatin (OXA) in human xenograft tumor cells (XhCRC) and protected XhCRC cells from cytotoxic therapy (OXA), indicating that fibroblasts may contribute to chemotherapeutic resistance via paracrine pathways.

**Fig. 1:**
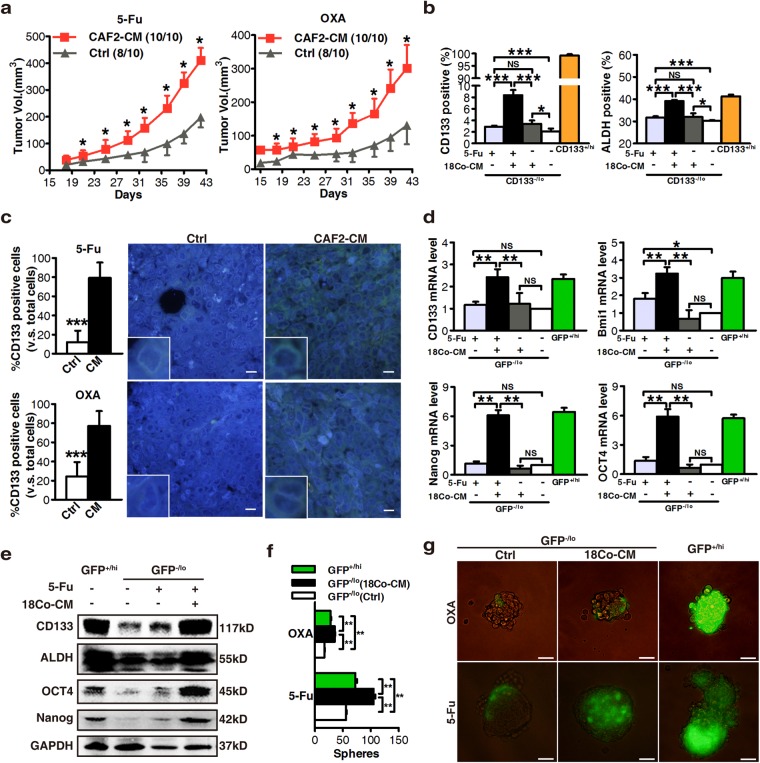
Fibroblasts endow differentiated CRC cells with stem cell-like properties and promote drug resistance via paracrine signaling. **a** Effects of CAF2-CM on the growth of CD133^−/lo^ XhCRC1 cells (5 × 10^5^) inoculated into NOD/SCID mice (*n* = 5) upon administration of 5-Fu or OXA. Tumor growth curves are shown. *P < 0.05. **b** CD133^−/lo^ SW620 cells treated with 18Co-CM in vitro were analyzed by flow cytometry to determine the expression of the stem cell maker CD133 and the functional stem cell factor Aldefluor (ALDH). *P < 0.05, **P < 0.01. **c** Paraffin-embedded sections of XhCRC1 xenografts were stained for CD133. Positive cells were quantified as a percentage of the total cells. *P < 0.05, **P < 0.01. Representative CD133 immunofluorescences of mice xenograft sections are shown. Scale bars, 10 μm. **d** mRNA levels of stem cell markers (*CD133*, *Nanog*, *OCT4*, and *Bmi1*) were quantified by quantitative RT-PCR in GFP^−/lo^ SW620 cells treated with or without 18Co-CM. **P < 0.01. **e** Immunoblot analysis of several stem markers (CD133, Nanog, OCT4, ALDH) in different treated GFP^−/lo^ SW620 cells. **f**, **g** Effects of 18Co-CM on the sphere-forming capacity of GFP^−/lo^ SW620 cells upon administration of 5-Fu or OXA, with GFP^+/hi^ cells as a positive control. **P < 0.01. Representative images are shown. Scale bars, 100 μm

Our previous study verified that CD133-positive CRC cells are enriched for CSCs, which are more resistant to chemotherapy than differentiated CD133-negative cells both in vitro and in vivo [8]. To determine the impact of fibroblasts on differentiated CRC cells (or non-CSCs), we purified CD133^+/hi^ and CD133^−/lo^ cells by fluorescence-activated cell sorting (FACS) and verified their phenotypes in CRC cells (Supplementary Figures S2a and b). In vitro sphere formation assays showed that the clonogenicity was significantly increased in fibroblast-derived CM-treated CD133^−/lo^ CRC cells (HT-29, SW620 [16], XhCRC) upon administration of either 5-Fu or OXA (Supplementary Figure S2c), suggesting that fibroblasts endow differentiated CRC cells with an enhanced self-renewal capacity through their secreted factors during chemotherapy. We further confirmed the promoting effects of CM on tumor growth in vivo using transplantation assays. Results showed that CM-treated CD133^−/lo^ CRC cells grew faster and generated larger tumors than CD133^−/lo^ cells with control medium during chemotherapy (5-Fu or OXA) (Fig. 1a, Supplementary Figures S2d–f). Collectively, these findings demonstrate that fibroblasts may contribute to enhanced chemotherapeutic resistance in differentiated CRC cells via paracrine pathways.

### Fibroblast-secreted factors endow differentiated CRC cells with stem cell-like properties

The above results from two CRC cell lines and one xenograft model showed that, during chemotherapy, CM from cultured fibroblasts (18Co) or patient tumor-derived CAFs promoted drug resistance in differentiated (CD133^−/lo^) CRC cells. To explore the underlying mechanisms of this process, we estimated effects of CM on the expression of two CRC CSC markers, CD133 and Aldefluor activity [2, 3, 17, 18]. CD133^−/lo^ SW620 cells treated with 18Co-CM showed the highest CD133 and Aldefluor activity upon 5-Fu treatment (Fig. 1b, Supplementary Figure S3a). Similarly, immunofluorescence analysis of the tumors from Fig. 1a showed that CAF2-CM-treated tumors possessed a higher percentage of CD133 positive cells compared with the control treated tumors (Fig. 1c). These results suggest that fibroblast-CM, along with chemotherapeutic treatment, promoted the development of a CSC phenotype in CD133^−/lo^ CRC cells.

To further test the above hypothesis, we infected SW620 cells with the viral vector TOP^−^GFP, a TCF/LEF reporter used to evaluate Wnt activity; that is frequently upregulated in CSCs [6]; and confirmed by genomic DNA PCR for *GFP* and quantitative PCR for *TCF* and *LEF* (Supplementary Figures S3b and c). Flow cytometry showed that 18Co-CM-treated GFP^−/lo^ (i.e., Wnt^−/lo^) SW620 cells acquired a higher percentage of GFP^+^ cells compared with the control medium (Supplementary Figure S3d), implying that fibroblasts stimulate a phenotypic reversion in differentiated (WNT^−/lo^) cells via paracrine mechanisms. In addition to phenotypic reversion, genes associated with stem cell functions were significantly elevated in WNT^−/lo^ cells at the mRNA and protein levels after treatment with 18Co-CM (Fig. 1d, e).

To investigate the functional consequences of phenotypic reversion, sphere-formation assays showed that 18Co-CM-treated GFP^−/lo^ cells generated more spheres in either 5-Fu or OXA compared with the control medium (Fig. 1f); importantly, the spheres contained more GFP^+^ cells (Fig. 1g). The above results showed that CAFs may induce differentiated CRC cells to restore their clonogenic and tumorigenic potential and to dedifferentiate into autonomous drug-resistant CSCs through paracrine signaling, thereby contributing to enhanced drug resistance.

### Exosomes contribute to the dedifferentiation of differentiated CRC cells and subsequent drug resistance

**Fig. 2:**
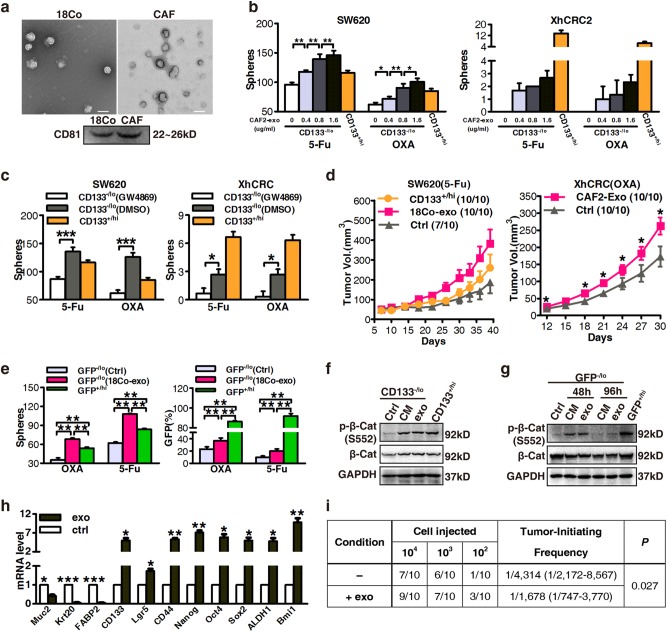
Exosomes contribute to the dedifferentiation of differentiated CRC cells and subsequent drug resistance. **a** Electron micrograph of exosomes isolated from 18Co cells and CAFs (top; scale bar, 100 nm) and immunoblotting analysis of the exosome marker CD81 (bottom). **b** The sphere-forming capacity of CD133^−/lo^ SW620 or XhCRC2 cells treated with indicated concentrations of exosomes during chemotherapy (5-Fu or OXA), with CD133^+/hi^ cells as the positive control. *P < 0.05, **P < 0.01. **c** CM derived from GW4869-pretreated fibroblasts was added to CD133^−/lo^ CRC cells in a sphere formation assay, and CD133^+/hi^ CRC cells were used as a positive control. *P < 0.05, ***P < 0.001. **d** Effects of exosomes on the growth of CD133^−/lo^ CRC cells (1 × 10^5^ SW620 cells or 4 × 10^5^ XhCRC2 cells) inoculated into immunocompromised mice (*n* = 5) upon administration of 5-Fu or OXA. Tumor growth curves are shown, and CD133^+/hi^ cells were used as a positive control in SW620 cells. *P < 0.05. **e** The sphere-forming capacity of GFP^−/lo^ SW620 cells with or without 18Co-exosomes during chemotherapy (5-Fu or OXA), with GFP^+/hi^ cells as a positive control. TOP-GFP expression of spheres was analyzed by flow cytometry. **P < 0.01. **f** Immunoblotting of total β-catenin and S552-phosphorylated β-catenin in CD133^−/lo^ SW620 cells after stimulation with 18Co-CM or exosomes, and CD133^+/hi^ CRC cells as positive control. **g** Immunoblotting of total β-catenin and S552-phosphorylated β-catenin in GFP^−/lo^ SW620 cells after stimulation with 18Co-CM or exosome for 48 h and in the absence of CM or exosome for another 48 h. **h** mRNA levels of several differentiation markers (*mucin2, cytokeratin 20, FABP2*) and CSC makers (*CD133*, *Lgr5, CD44, Nanog, OCT4, SOX2, ALDH1* and *Bmi1*) in exosome-treated spheres in CD133^−/lo^ XhCRC cells. *P < 0.05. **P < 0.01, ***P < 0.001. **i** Tumor-initiating frequency of exosome-treated CD133^−/lo^ XhCRC cells in NOD/SCID mice

Exosomes are emerging as novel secreted regulators in cell–cell communication. Therefore, we investigated the role of exosomes derived from fibroblasts in drug resistance in differentiated CRC cells. We first separated exosomes from fibroblast-CM using a total exosome isolation kit, and confirmed their structural features by phase-contrast electron microscopy and immunoblotting of the known exosome marker CD81 (Fig. 2a). We labeled exosomes with DiI, a membranal fluorescent carbocyanine dye, and found that Dil-labeled exosomes derived from 18Co cells were taken up by SW620 cells after 12 h co-incubation (Supplementary Figure S4a). To test whether fibroblast-derived exosomes could induce drug resistance in differentiated CRC cells, we treated CD133^−/lo^ CRC cells with purified exosomes instead of CM, and found that both SW620 and XhCRC CD133^−/lo^ cells treated with exosomes generated more spheres in a dose-dependent manner (Fig. 2b). We therefore treated fibroblasts (18Co and CAFs) with GW4869, a specific neutral sphingomyelinase inhibitor [19] that blocks exosome release (Supplementary Figures S4b and c), and then obtained the CM (exosome-depleted CM), which was added to CD133^−/lo^ CRC cells treated with either 5-Fu or OXA. The sphere formation assay demonstrated that exosome-depleted CM had diminished sphere-promoting effects on CD133^−/lo^ CRC cells compared with the vehicle-pretreated CM (Fig. 2c), suggesting that exosomes were causally involved in the dedifferentiation of differentiated CRC cells during chemotherapy. To confirm that the fibroblast-secreted exosomes mediated the observed effects rather than other soluble factors, we also adopted an ultracentrifugation approach to isolate exosomes. Similar to kit-purified exosomes, CM-pellet-treated CD133^−/lo^ SW620 cells formed more spheres compared with control pellets, whereas the exosome-depleted supernatant from 18Co-CM showed a slight but negligible increase (Supplementary Figure S4d). In addition, in vivo experiments showed that CD133^−/lo^ CRC cells treated with purified exosomes, generated faster-growing and larger tumors (Fig. 2d, Supplementary Figure S4e) than control groups during chemotherapy. These data clearly show that fibroblast-derived exosomes caused differentiated CRC cells to be more drug resistant. More importantly, exposure of GFP^−/lo^ SW620 cells to purified exosomes induced a higher clonogenic capacity and Wnt reporter activity (Fig. 2e). In differentiated CRC cells, stimulation with CM or purified exosomes strongly induced β-catenin stability through an increase in the phosphorylation of β-catenin on Ser 552 (Fig. 2f, g), which is associated with enhanced transcription of Wnt target genes [20]. Moreover, after removing the CM or exosomes for 48 h, the phosphorylation of β-catenin on Ser 552 vanished (Fig. 2g). Furthermore, real-time PCR revealed that differentiation makers (mucin2, cytokeratin 20, FABP2) were downregulated in the exosome-treated CD133^−/lo^ XhCRC cells, whereas CSC makers (CD133, Lgr5, CD44, Nanog, Oct4, Sox2, ALDH1, and Bmi1) were increased (Fig. 2h). In addition, limiting dilution assays (LDAs) demonstrated a higher tumor-generating capacity in exosome-treated CD133^−/lo^ XhCRC cells compared with control cells (Fig. 2i, Supplementary Figure S4f). Together, these results demonstrated that CAFs-secreted exosomes may contribute to the induction of dedifferentiation of differentiated cells thus promoting drug resistance in CRC.

### Wnts are secreted in fibroblast-derived exosomes and are correlated with poor prognosis

**Fig. 3:**
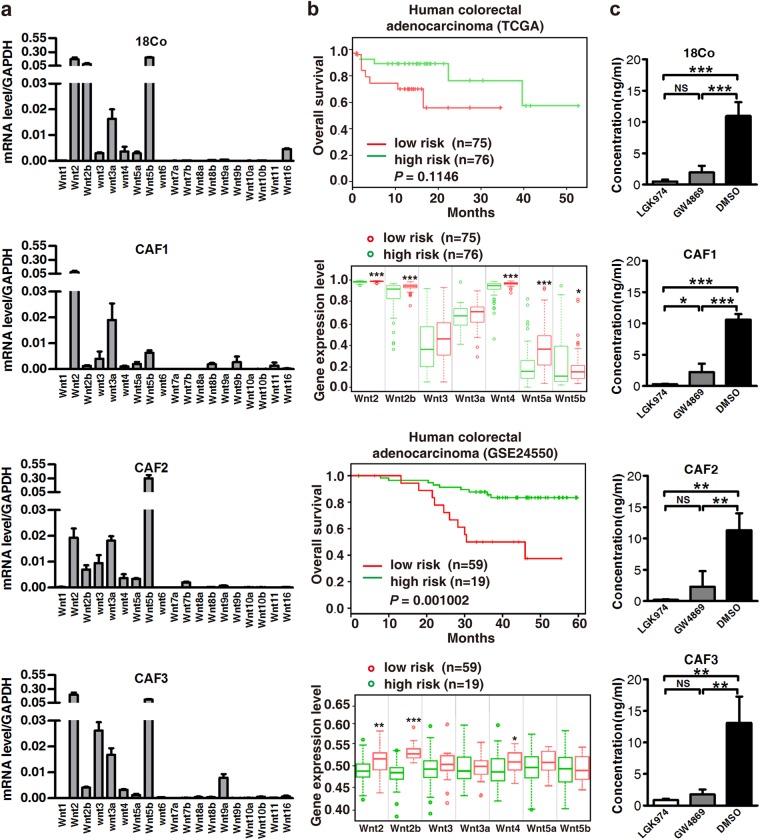
Wnts are secreted in fibroblast-exosomes and predict a poor prognosis. **a** Real-time PCR analysis of RNA from fibroblasts for Wnt mRNA expression. *Wnt2, Wnt2b, Wnt3, Wnt3a, Wnt4, Wnt5a* and *Wnt5b* were expressed in all four fibroblasts (18Co cells, CAF1, CAF2, and CAF3). **b** Kaplan–Meier curves showing that high expression of *Wnt2, Wnt2b, Wnt3, Wnt3a, Wnt4, Wnt5a*, and *Wnt5b* in two microarray data sets are positively associated with poor patient survival (*P* = 0.115 in TCGA and *P* = 0.001 in GSE24550). **c** Enzyme-linked immunosorbent assay (ELISA) to detect Wnt3a in exosomes from LGK974- or GW4869-treated 18Co cell-derived CM. **P < 0.01

It has been previously shown that Wnt/β-catenin signaling activity is a functional marker for CSCs, plays a major role in maintaining stemness in colon cancer and is regulated by the extrinsic microenvironment [6, 21]. Wnts are critical ligands to activate Wnt pathways, leading to the transcriptional activation of TCF/LEF to drive the expression of Wnt target genes. Recent studies demonstrated that Wnts are secreted in exosomes [14, 22]. To identify the exosomal Wnts responsible for drug resistance in differentiated CRC cells, we first evaluated Wnt expression in fibroblasts. Real-time PCR analysis indicated that, of the 19 mammalian Wnt proteins [23], Wnt2, Wnt2b, Wnt3, Wnt3a, Wnt4, Wnt5a, and Wnt5b were stably expressed in 18Co cells and three CAFs derived from three patient tumors (Fig. 3a). CRC cells, in contrast, expressed a different pattern and much lower levels of Wnts (Supplementary Figure S5). Moreover, examination of Wnt gene expression by *SurvExpress* from two microarray data sets demonstrated that increased expression of Wnt genes was positively correlated with shortened patient survival in CRC (Fig. 3b). Of the above seven Wnts, Wnt3a showed modest and consistent expression in all four fibroblasts, therefore we chose Wnt3a for further investigation. As shown in Fig. 3c, the proportion of exosome Wnt3a was significantly decreased in CM derived from 18Co pretreated with LGK974, a porcupine inhibitor that blocks Wnt secretion [24, 25] (Fig. 3c). In addition, exosome secretion inhibitor GW4869 caused the same decrease in exosome Wnt3a (Fig. 3c), further affirming that Wnts are secreted in exosomes.

### Exosomal Wnts protect differentiated CRC cells against chemotherapy

**Fig. 4:**
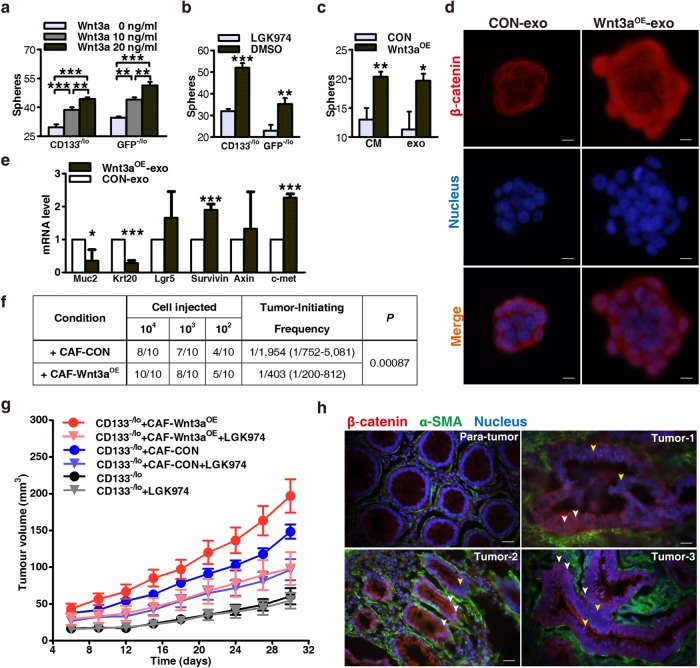
Exosomal Wnts protect differentiated CRC cells against chemotherapy. **a** Effects of exogenous Wnt3a protein on the sphere-forming capacity of differentiated SW620 cells (CD133^−/lo^ or GFP^−/lo^ cells) during chemotherapy (OXA). **P < 0.01, ***P < 0.001. **b** Exosomes were derived from LGK974-pretreated 18Co cells and then added to differentiated SW620 cells (CD133^−/lo^ or GFP^−/lo^ cells). Their effects on sphere-forming capacity were estimated upon treatment with OXA. **P < 0.01, ***P < 0.001. **c** CM and exosomes were made from Wnt3a-overexpressing CAF3 and added to differentiated XhCRC cells (CD133^−/lo^ XhCRC2 cells). Their effects on the sphere-forming capacity of differentiated XhCRC cells were estimated upon treatment with OXA. *P < 0.05, **P < 0.01. **d** Representative images of spheres from **c** stained with β-catenin (red). Nuclei were stained with 4,6-diamidino-2-phenylindole (DAPI; blue). Wnt3a^OE^-exosome-treated CD133^−/lo^ XhCRC2 cells showed more nuclear β-catenin staining compared with the control. Scale bars, 10 µm. **e** mRNA levels of several differentiation markers (*mucin2, cytokeratin 20*) and Wnt target genes (*Lgr5, Survivin, Axin* and *c-Met*) in exosome-treated spheres from **c**. *P < 0.05, ***P < 0.001. **f** Tumor-initiating frequency of exosome-treated CD133^−/lo^ XhCRC cells in NOD/SCID mice. **g** CD133^−/lo^ XhCRC2 cells (6 × 10^4^) were injected (*n* = 5 per group) with or without 6 × 10^4^ Wnt3a^OE^-CAF3 or control CAF3 into NOD/SCID mice, and all mice were intraperitoneally treated with OXA and orally fed with LGK974 or control vehicle. Tumor growth curves are shown. Data are presented as the mean ± SEM. **h** Immunofluorescence co-staining for α-SMA and β-catenin in frozen sections of human colorectal cancer specimens. Cancer cells positive for nuclear β-catenin (red) showed an intimate localization close to α-SMA-positive fibroblasts (green). Nuclei were stained with DAPI (blue). Scale bars, 20 µm

Though little information links Wnt3a to drug responses, previous studies have reported that Wnt3a is a critical ligand to activate Wnt pathways via paracrine signaling [13, 26, 27]. We confirmed that exogenous recombinant Wnt3a increased the clonogenic capacity of differentiated (Wnt^−/lo^ and CD133^−/lo^) CRC cells in a dose-dependent manner (Fig. 4a). Conversely, blocking Wnt secretion using exosomes derived from 18Co cells pretreated with porcupine inhibitor LGK974 suppressed their clonogenicity-promoting effects, as well as causing a concomitant sharp decline in Wnt activity and the percentage of CD133-positive cells (Fig. 4b, Supplementary Figure S6a). To further investigate the impact of Wnts in this process, we established CAFs that overexpressed Wnt3a (Supplementary Figure S6b). We made CM from Wnt3a^OE^ CAFs (Wnt3a^OE^-CM) and isolated the resultant exosomes (Wnt3a^OE^-exosomes); CM or exosomes derived from vector-infected CAFs were used as the control. Enzyme-linked immunosorbent assay (ELISA) and immunoblotting confirmed high Wnt3a expression in exosomes derived from Wnt3a^OE^-CAFs (Supplementary Figures 4c and d). Wnt3a^OE^-CM and Wnt3a^OE^-exosomes were then added to CD133^−/lo^ XhCRC cells and sphere formation assays were performed. Both Wnt3a^OE^-CM and Wnt3a^OE^-exosomes exhibited enhanced clonogenicity-promoting effects compared with controls during chemotherapy (Figure 6c). Moreover, stimulation with Wnt3a^OE^-CM or Wnt3a^OE^-exosomes strongly increased nuclear β-catenin localization in CD133^−/lo^ XhCRC spheres (Fig. 4d, Supplementary Figure S6e). Consistent with the above findings, real-time PCR confirmed that differentiation makers (mucin2 and cytokeratin 20) were downregulated in the Wnt3a^OE^-exosome-treated CD133^−/lo^ XhCRC cells, whereas Wnt target genes (those encoding Lgr5, Survivin, Axin, and c-Met) were increased (Fig. 4e). Furthermore, LDAs demonstrated a higher tumor-generating capacity in Wnt3a^OE^-exosome-treated CD133^−/lo^ XhCRC cells compared with control cells (Fig. 4f, Supplementary Figure S6f). Taken together, these results indicate that fibroblast-secreted exosomal Wnts enhance Wnt activity and can reactivate features of stemness in differentiated CRC cells.

Next, we explored the inhibition of Wnt-dependent dedifferentiation as a potential therapeutic strategy in CRC. Female NOD/SCID mice received CD133^−/lo^ XhCRC cells alone, or co-implanted with Wnt3a-overexpressed or control CAFs. Upon administration of the chemotherapeutic agent OXA, recipient mice were treated with LGK974 or vehicle. We found that tumor cells co-implanted with Wnt3a^OE^ CAFs exhibit elevated drug resistance in recipient mice, which appeared as substantially faster tumor growth and larger tumor volume compared with co-implanted control CAFs or without CAFs (Fig. 4g, Supplementary Figure S6g). Surprisingly, this tumor-promoting effect was markedly suppressed in tumor-bearing mice treated with LGK974 (Fig. 4g, Supplementary Figure S6g), suggesting that inhibiting Wnt secretion can disrupt drug resistance in differentiated CRC cells. In addition, in samples of primary colon cancer, we observed a clear co-localization between cells expressing α-smooth muscle actin (α-SMA) and tumor cells displaying nuclear β-catenin (white arrows in Fig. 4h). These data clearly showed that fibroblast-derived exosomal Wnts primed differentiated CRC cells acquiring CSC phenotype and then become more drug resistance.

## Discussion

**Fig. 5:**
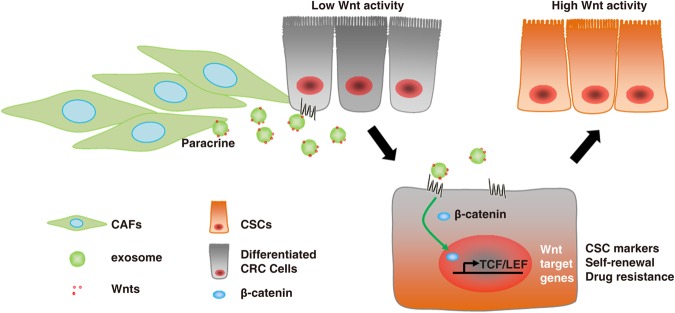
Schematic representation of the proposed model. Fibroblasts reside close to cancer cells in colorectal cancer (CRC) tissue. Fibroblasts secrete exosomal Wnts that stimulate differentiated CRC cells to restore their cancer stem cell (CSC) characteristics, including the expression of CSC markers and elevated Wnt activity; this contributes to drug resistance

In this study, by focusing on subpopulations of cancer cells (i.e., cancer cell heterogeneity) and the tumor microenvironment, we presented evidence that stromal fibroblasts secrete exosomes, that promote the phenotypic reversion and functional acquisition of CSC properties in differentiated CRC cells by carrying Wnt ligands to activate Wnt signaling, which contributes to chemoresistance (summarized in Fig. 5).

Consistent with previous studies [28, 29], our analysis demonstrated that CM derived from both fibroblasts and CAFs, stimulated chemoresistance in CRC cells, suggesting that CAFs contribute to drug resistance via secreted factors. Nevertheless, how stromal cells contribute to chemoresistance remains unknown. Like most other tumors, CRC is heterogeneous and contains both undifferentiated CSCs and differentiated tumor cells. CSCs have been found to be inherently drug-resistant and to possess developmental plasticity, as they can differentiate into more mature progeny [30–32]. And differentiated cancer cells can convert into a stem-like state [30, 33, 34]. Nevertheless, the possibility that fibroblast-derived secreted factors may mediate chemoresistance by conferring CSC properties or “reprogramming” differentiated CRC cells to CSCs has not been prospectively addressed in CRC. Our data indicated that several phenotypically differentiated CRC subpopulations, such as CD133^−/lo^, ALDH^−/lo^, and TOP-GFP^−/lo^ cells, when treated with chemotherapeutic agents, acquired both phenotypic markers and importantly, functional properties of CSCs. Indeed, upon treatment and in the presence of fibroblast-derived CM, purified differentiated CRC cell subpopulations acquired substantially higher percentages of cells positive for CRC CSC makers both in vitro and in vivo. Furthermore, these phenotypically reprogrammed CRC cells became more drug resistant, and exhibited enhanced sphere-formation capability and higher tumorigenicity in the presence of CM. One recent study suggested that CAFs may contribute to drug resistance in CRC cells through maintaining the pre-existent CSC pool [7]. Our previous study verified that CM derived from fibroblasts (18Co) or CAFs enhanced sphere-formation capability and tumorigenicity in CD133^+/hi^ CSCs [8], suggesting that both mechanisms (i.e., maintaining the CSC pool and reprogramming differentiated CRC cells to CSCs) may cooperate to confer chemoresistance. Recent studies have suggested that the developmental potential of CSCs may be regulated by both genetic and epigenetic mechanisms [31, 35]. We analyzed the expression of several “stemness” genes including CD133, Nanog, OCT4, and Bmi1, and found that phenotypically differentiated cell populations treated with fibroblast-derived CM, under chemotherapeutic stress, expressed higher protein and mRNA levels of these molecules, supporting that dedifferentiation may indeed have occurred during chemotherapy. Of interest, although differentiated cancer cells can dedifferentiate into CSCs, under normal (or unperturbed) conditions, they manifest limited plasticity, seldom converting into CSCs; however, under “induced” (or perturbed) conditions, such as chemotherapy, radiation, hypoxia, and inflammatory mediators, differentiated cancer cells might acquire enhanced phenotypic and functional plasticity [30, 33, 34, 36]. In consistence, we found that, without chemotherapeutic agents, fibroblast-derived CM showed a slight or even no effect on those CSC makers in differentiated cells.

In recent years, the role of exosomes in cancer as mediators of cell–cell communication within the microenvironment has gained increasing attention. Exosomes may act as natural vehicles for delivering protein, mRNA, or microRNA to recipient cells [11, 37–39] to mediate their biological and pathological functions [40]. A variety of cells may secrete exosomes. For example, melanoma cells secrete exosomes to “educate” bone marrow progenitor cells and promote metastasis [41]. In another study, a panel of cancer cell lines was shown to produce exosomes, which help to induce differentiation of fibroblasts toward a myofibroblastic phenotype [42]. Our studies identified exosomes as the paracrine entities released from fibroblasts that are involved in the dedifferentiation of differentiated CRC cells. In support of this, exosomes can be purified from the CM derived from both 18Co cells and primary CAFs. In addition, purified exosomes directly promoted the acquisition of the CSC phenotype in differentiated (i.e., CD133^−/lo^, ALDH^−/lo^, or TOP-GFP^−/lo^) CRC cells during chemotherapy. Furthermore, purified exosomes promoted sphere-formation and tumorigenic capability in the differentiated CRC cells. Finally, inhibition of exosome release blocked the above effects. In the present study, we showed that exosomes derived from fibroblasts can promote drug resistance by reprogramming differentiated CRC cells toward CSCs.

Wnt/β-catenin signaling plays a major role in maintaining stemness in colon cancers. In a similar manner to that of normal intestinal stem cells, Wnt activity is not merely a cell-intrinsic feature that can be used to define the colon CSC population, but is also regulated by the extrinsic microenvironment [6, 21]. Wnt proteins bind to Frizzled/LRP receptors on target cells leading to β-catenin accumulation and nuclear translocation to activate Wnt signaling [12]. Recent studies have shown that exosomes are ideal vehicles for the delivery of insoluble hydrophobic Wnt proteins [22]. Saha et al. [14] showed that macrophage-derived exosomal Wnts are required for the regeneration of intestinal stem cells in response to radiation. Raphael et al reported that, exosomal Wnts originating from CSCs is essential for itself clonogenicity and proliferation in lymphoma [43]. Luga et al. [40] found that fibroblast-derived exosomal Wnts can enhance cell mobility and metastasis in breast cancer; and Chen et al. [44] also demonstrated that fibroblasts promote breast cancer cell metastasis through exosomal Wnt10b. However, whether exosomal Wnts involved in dedifferentiation of differentiated cells and its association with drug resistance in CRC is not clear. In this study, we found that Wnts are indeed secreted in fibroblast-derived exosomes and that these exosomal Wnts can enhance Wnt activity and stimulate the CSC phenotype in differentiated CRC cells, leading to increased drug resistance in vitro and in vivo. Inhibition of Wnt secretion by the porcupine inhibitor LGK974 can disturb this process. Our results showed that the inhibition of exosomes generating by GW4869 only partially reduced the secretion of Wnts, implying that Wnts possess other secretion routes. In addition, we observed a clear co-localization between fibroblasts and tumor cells displaying nuclear β-catenin in primary colon cancer samples. Together, these findings show a close relationship between CSC properties, drug resistance, and the tumor environment in CRC.

In conclusion, our results showed that fibroblasts can reprogram differentiated cells to CSCs by transferring exosomal Wnts thus contributing to chemoresistance in CRC. Novel therapeutics targeting preventing the exosomal transfer of Wnts from stromal cells should be developed to assist in preventing tumor recurrence.

## Materials and methods

### Cell culture

The CCD-18Co cell line was purchased from ATCC (Manassas, VA, USA; CRL-1459) and cultured in Dulbecco’s modified Eagle’s medium (DMEM; Gibco; Waltham, MA, USA). HT-29 and SW620 cell lines were purchased from the cell bank of Chinese Academy of Sciences (Shanghai, China). HT-29 cells were cultured in McCoy’s 5a medium (Gibco), and SW620 cells were cultured in DMEM (fetal bovine serum (FBS), Gibco). All cell lines were supplemented with 10% FBS (Gibco) and incubated at 37 **°**C with 5% CO_2_ in a humidified cell culture incubator. All cell lines were routinely tested for mycoplasma contamination before use.

### Isolation of colorectal CAFs

Primary human colorectal tumor specimens were collected under IRB-approved guidelines and with informed patient consent at the Tongji Hospital of Huazhong University of Science and Technology, Wuhan, China (IRB ID: 20141106). All patients have complete medical records. Detailed clinic information is summarized in Supplementary Table 1. None of the patients received preoperative chemoradiation treatments. CAFs were obtained from human CRC tissues as described previously [7, 45]. In brief, fresh specimen tissue was mechanically dissociated with sterilized scalpels and scissors, followed by 1–2 h incubation in DMEM/F12 containing 1.5 mg/ml collagenase IV (Gibco), 20 μg/ml hyaluronidase (Sigma Aldrich, St. Louis, MO, USA), 500 U/ml penicillin, 500 mg/ml streptomycin, and 1.25 mg/ml amphotericin B at 37 °C. The specimen was filtered through sterile 100-μm strainers to obtain single-cell suspensions. Red blood cells were then eliminated with a hypo-osmotic red blood cell lysis buffer (BioLegend, San Diego, CA, USA). Isolated single cells of fresh specimens were stained with EpCAM and subjected to FACS (Aria II, BD Biosciences, San Jose, CA, USA) to purify EpCAM-negative CAFs. Cells were then cultured in DMEM with 10% FBS(Gibco). CAFs were confirmed by immunofluorescence with fibroblast markers such as vimentin, FAP-1, and α-SMA, and were also negative for several epithelial cell markers including EpCAM, CDX2, and CK7. All primary fibroblasts were used by the tenth passage.

### Establishment of human CRC xenograft tumors

XhCRC were routinely maintained in female NOD/SCID mice [46, 47]. Fresh human tissues acquired from surgical specimens from primary human colorectal tumor patients were dissociated into single cells as described above, and then injected in 50% Matrigel (v/v with phosphate-buffered saline (PBS), BD Biosciences) SC in female NOD/SCID mice. After the tumors matured, they were harvested, human CRC cells were purified, and the cells were injected SC in 50% Matrigel in female NOD/SCID mice. This process was repeated every generation (~ 2 months). All the clinical information is listed in Supplementary Table 1.

### CM preparation

CM was derived from 18Co cells or CAFs. After cell cultures reached 90% confluency, cells were washed with PBS to eliminate all traces of serum in the cultures and incubated with fresh DMEM/F12 at 37 °C, and 5% CO_2_. After 2 h, CM was collected and centrifuged at 2000 × *g* for 10 min at 4 °C. Supernatant was filtered through a 0.22-µm filter (Millipore, Billerica, MA, USA) to remove the cellular debris.

### Exosome purification using exosome isolation reagent

Exosome purification from CM was performed using a Total Exosome Isolation Kit (Invitrogen, Carlsbad, CA, USA) according to the manufacturer’s protocol. In brief, 0.5 ml total exosome isolation reagent was added to 1 ml filtered CM and mixed well by inverting. After being incubated overnight, the mixture was centrifuged at 12,000 × *g* for 70 min at 4 °C and the supernatant was removed. Exosome pellets were suspended in a convenient volume of DMEM/F12. The concentration of exosomes was assessed using Pierce bicinchoninic acid (BCA) Protein Assay (Thermo Fisher Scientific, Waltham, MA, USA) according to the manufacturer’s instructions. The concentration of purified exosomes was 40 µg/ml for in vitro assays and 4 µg/ml for in vivo assays.

### Exosome purification by differential centrifugation

Exosomes were isolated from fibrobalst-derived CM as previously described [48]. In brief, CM was centrifuged at 2000 × *g* for 20 min and 10,000 × *g* for 40 min to remove cell debris and other types of vesicles, and then subjected to ultracentrifugation at 100,000 × *g* for 70 min. The final exosome pellet was re-suspended in DMEM/F12 and the supernatant, which contained soluble factors, was retained.

### Transmission electron microscopy

Exosome samples were prepared by mixing with an equal volume of 4% paraformaldehyde (PFA) and deposited onto Formvar-carbon-coated copper grids. Grids were stained with 2% uranyl acetate for 10 min and air dried. The samples were observed in a FEI Tecnai T20 transmission electron microscope (TEM; FEI Tecnai 20; Philips, Amsterdam, Netherlands) at an accelerating voltage of 160 kV. Images were taken at 25,000 × magnifcation. The size of exosomes was measured based on three to five different pictures using the TEM software.

### Sphere-formation assays

Basic procedures for sphere-formation assays were performed as previously described [49]. Cells were grown in standard sphere-forming medium (serum-free DMEM/F12 (1:1) supplemented with 1 × B27 serum substitute (Gibco), 20 ng/ml human recombinant epidermal growth factor (Sigma Aldrich) and 20 ng/ml basic fibroblast growth factor (Sigma Aldrich). CRC cells were plated at ~ 300–500 cells/well in 24-well ultra-low attachment plates (Corning Inc., Corning, NY, USA) upon administration of 5-Fu (1 μm) or OXA (1 μm). CM (200 μl) or exosomes (0.8 μg/ml) were added every 3 days. 18Co-derived CM or exosomes were used in CRC cell lines and CAF-derived CM or exosomes were used in XhCRC cells. Spheres that arose within 5–14 days are shown as clonogenicity (%) or sphere numbers in the figures. Spheres were visualized via light microscopy (Olympus CKX41; Olympus, Tpkyo, Japan).

### In vivo mouse assays

All animal studies were performed under the guidelines and protocols approved by the Institutional Animal Care and Use Committee of Tongji Medical College, Huazhong University of Science and Technology (IACUC No.: S652). Mice were sorted randomly to each experimental and control group. In all experiments, cell viability was confirmed through a trypan blue exclusion test, and cells were re-suspended in 100 ml PBS mixed with Matrigel at 1:1 ratio. CRC cell lines (HT-29 and SW620 cells) were SC implanted into 4-week-old female BALB/c-nu mice, and XhCRC cells were implanted into 4-week-old female NOD/SCID mice. CM (200 μl) or exosomes (0.4 μg in 100 μl DMEM/F12) were SC injected around the tumor injection sites every 2 days.18Co-derived CM or exosomes were used for CRC cell lines and CAF-derived CM or exosomes were used for XhCRC cells studies. For the co-injected tumor study, 18Co cells were pretreated with MMC at the concentration of 1 μg/ml for 1 h. HT-29 cells were then implanted with or without equal MMC-pretreated 18Co cells into the flanks of nude mice. Simultaneously, all the mice were intraperitoneally treated with a chemotherapeutic agent, 5-Fu (Sigma Aldrich; 100 mg/kg body weight) or OXA (Sigma Aldrich; 10 mg/kg body weight) once a week. For limiting dilution assays, 10,000, 1000, and 100 CD133^−/lo^ CRC cells were implanted into 4-week-old female NOD/SCID mice, which then treated with or without CAF-derived exosomes upon administration of OXA. For the co-injected limiting dilution assays, 10,000, 1000, and 100 CD133^−/lo^ CRC cells were implanted with equal MMC-pretreated Wnt3a-overexpressing CAFs or control CAFs into 4-week-old female NOD/SCID upon administration of OXA. Tumor-initiating frequency and statistical significance were evaluated with the Extreme Limiting Dilution Analysis (ELDA) “limdil” function (http://bioinf.wehi.edu.au/software/elda/index.html). In the Wnt secretion inhibition assay, mice were treated with LGK974 (3 mg/kg body weight, dissolved in 0.5% carboxymethylcellulose and 0.5% Tween 80) or vehicle (0.5% carboxymethylcellulose and 0.5% Tween 80 only) daily. Tumors were monitored, and tumor sizes were assessed three times per week after the tumors were palpable using caliper measurements. Measurement in mouse was performed blinded (the investigator had no knowledge of the treatment at that point in time). Tumor volumes were calculated using formula (length × width^2^)/2. After the mice were killed, tumors were removed and weighed to evaluate tumor development. Three to five mice were used for each experimental condition.

### FACS

FACS was performed according to the manufacturer’s instructions using a FACS Aria II Cell Sorter (BD Biosciences), followed by flow cytometric analysis using Diva software (BD Biosciences). To enrich for EpCAM-negative CAFs, single cells from CRC tumor specimens were labeled with Allophycocyanin (APC)-conjugated monoclonal antibody against EpCAM (Miltenyi Biotec, Bergisch Gladbach, Germany). Generally, the bottom 5–10% negative population (i.e., EpCAM-) was selected. To enrich for CD133^−/lo^ cells, single SW620 and HT-29 cells were labeled with phycoerythrin (PE)-conjugated monoclonal antibody against CD133 (Miltenyi Biotec). For the negative population, generally the bottom 10–20% cells (i.e., CD133^−/lo^) were purified out for functional assays. For the positive population, only the top 10–20% PE-bright (i.e., CD133^+/hi^) cells were collected. To purify EpCAM^+^CD133^−/lo^ cells from xenograft tumors, we co-stained XhCRC cells with antibody against CD133 (PE) and EpCAM (APC). Generally, 10–20% EpCAM^+^CD133^−/lo^ cells and 10–20% EpCAM^+^CD133^+/hi^ cells were purified.

### ALDEFLUOR assays

Cellular ALDH activity was measured using the ALDEFLUOR assay kit (Stem Cell Technologies, Vancouver, Canada) according to the manufacturer’s protocol [50]. After treatment, the cells were suspended in ALDEFLUOR assay buffer containing the ALDH substrate (1 μm per 1 × 10^6^ cells) and incubated for 30 min at 37 °C. As a negative control, 50 nmol/l diethylaminobenzaldehyde, a specific ALDH inhibitor, was added to the cell suspension.

### Immunofluorescence

Sorted cells were cultured in glass-bottomed Petri dishes in a monolayer overnight, and spheres were plated on six-well plates and subsequently fixed with 4% PFA for 10 min at room temperature. For CRC specimens, after being fixed with 4% PFA, the tissue was placed into Tissue-Tek OCT (SaKura Finetek USA) and sectioned into 8 μm slices. After being permeabilized with 0.025% Triton X-100 for 10 min, cells or tissues were blocked with 5% bovine serum albumin (BSA) in PBS for 1 h at room temperature. For paraffin-embedded XhCRC specimens, tissue sections were first heated in citrate buffer (pH 6.0) at 100 °C for 15 min in a microwave oven for immunostaining antigen retrieval and then blocked with 0.05% (w/v) BSA in PBS for 20 min. Primary antibodies were diluted at a ratio of 1:50 in 5% BSA. After being incubated overnight at 4 °C, cells were rinsed with PBS and incubated with a fluorophore-conjugated secondary antibody (diluted 1:50 in 5% BSA) for 2 h at room temperature. Samples were stained with 4′,6-diamidino-2-phenylindole (DAPI; Sigma Aldrich) and mounted with Anti-fade Mountant (Thermo Fisher Scientific) before being visualized via fluorescence microscopy (Olympus BX53 or CKX41) or confocal microscope (Olympus FV1000).

### RNA expression analysis

PCR, reverse transcription PCR and quantitative real-time PCR analyses were performed as previously described [51]. For PCR, genomic DNA was purified from GFP^+^ and GFP^−^ SW620 cells using the Universal Genomic DNA Extraction Kit (TaKaRa, Shiga, Japan). RNA was extracted using Trizol (TaKaRa) according to the manufacturer’s protocol, and cDNA was synthesized using PrimeScript RT Master Mix (TaKaRa). qPCR was performed using SYBR green reagents (TaKaRa) on an ABI PRISM 7300 Sequence Detection System (Applied Biosystems, Foster City, CA, USA). All primers are listed in Supplementary Table 2.

### Dil-labeled exosome transfer

Purified exosomes derived from 18Co cells were labeled with the lipophilic fluorescent carbocyanine dye, DiI (Santa Cruz Biotecnology, Dallas, TX, USA) [52]. Exosomes were suspended in 100 μl PBS incubated with 1 µl Dil for 15 min at 37 °C, washed twice to remove excess dye, and incubated with SW620 cells at 37 °C overnight. Dil-labeled exosome transfer was assessed using a fluorescence microscope (Olympus CKX41) by evaluating red cells.

### Inhibition of exosome release

To further validate the effects of exosomes derived from 18Co or CAFs, exosome release was suppressed using a specific inhibitor for neutral sphingomyelinase 2-GW4869 (Sigma Aldrich) [19]. CAFs or 18Co cells were plated in 10-cm dishes and incubated with GW4869 (10 μm) for 24 h. After incubation, the culture medium, including GW4869, was washed, and fresh culture medium was added, finally, CM was harvested as described above.

### Lentiviral transfection

The TCF1/LEF1 reporter driving expression of GFP (TOF-GFP) was purchased from SBO Medical Biotechnology Company (Shanghai, China). Lentiviral clones overexpressing Wnt3a and control vector were purchased from GeneChem Company (Shanghai, China). Using Lipofectamine 2000 (Invitrogen), SW620 cells were infected with the TOP-GFP lentivirus at multiplicity of infection (MOI) = 25, and CAFs were infected with Wnt3a-overexpressing lentivirus at MOI = 25 according to the manufacturer’s instructions.

### Immunoblotting

Cells and exosomes were lysed in radioimmunoprecipitation assay buffer (Sigma Aldrich) and protein concentration was determined using BCA assay (Thermo Fisher Scientific). The proteins were then separated by sodium dodecyl sulfate polyacrylamide gel electrophoresis. After being transferred onto polyvinylidene difluoride membranes, separate proteins in bands were blocked with 5% BSA in Tris-buffered saline for 1 h and probed with primary antibodies overnight at 4 °C. After washing, membranes were incubated with secondary antibodies for 1 h at room temperature and visualized with enhanced chemiluminescence reagents (Thermo Fisher Scientific).

### ELISA

Wnt3a secretion in exosomes was determined using an ELISA kit according to the manufacturer’s instructions (PeproTech, Rocky Hill, NJ, USA). Biotin-conjugated antibodies against Wnt3a (PeproTech) were used for ELISA. Purified exosomes (40 μg/ml) were used for each assay.

### Bioinformatics analyses

The clinical relevance of variable Wnt expression was assessed using publicly available data from the Cancer Genome Atlas (TCGA) and Gene Expression Omnibus (GEO); accession number: GSE24550) by *SurvExpress*, an online biomarker validation tool and database [53].

### Antibodies

The following antibodies were used for immunofluorescence: anti-Vimentin (Cell Signaling Technology, Danvers, MA, USA, 5741, 1:100), anti-FAP-1 (Abcam, Cambridge, UK, ab53066, 1:100), anti-α-SMA (Dako, Fisher City, CA, USA, M0851, 1:100,), anti-CDX2 (Epitomics, 2475-1, 1:100), anti-CK7 (Cell Signaling Technology, 4465, 1:100), anti-EpCAM (Miltenyi Biotec, 130-098-793, 1:50), anti-CD133 (Abcam, ab16518, 1:50), Alexa Fluor488-conjugated secondary antibodies (Jackson Immuno Research, West Grove, PA, USA, 1:50), PE-conjugated secondary antibodies (Dako, 1:50), and Cy3-conjugated secondary antibodies (Santa Cruz Biotechnology, 1:50). The following antibodies were used for immunoblotting: anti-CD81 (Santa Cruz Biotechnology, SC-7637, 1:500), anti-GAPDH (Abcam, ab9484, 1:1,000), anti-CD133 (Miltenyi Biotec, 130-092-395, 1:250), anti-Nanog (Cell Signaling Technology, 4903, 1:1000), anti-OCT4 (Cell Signaling Technology, 2890, 1:1000), anti-EphB2 (R&D Systems, AF467, 1:2000), anti-ALDH (Santa Cruz Biotechnology, sc-374076, 1:1000). anti-phospho-β-catenin (Cell Signaling Technology, Ser 552, 9566 s, 1:500) anti-β-catenin (Cell Signaling Technology, 8480 s, 1:1,000), anti-Wnt3a (Santa Cruz Biotechnology, sc136136, 1:200), and horseradish peroxidase-conjugated secondary antibodies (Santa Cruz Biotechnology 1:10,000).

### Reagents

Recombinant mouse Wnt3a (R&D Systems, 1324-WN) was dissolved in PBS containing 0.2% BSA. For in vitro assays, LGK974 (MedChem Express, Monmouth Junction, NJ, USA, HY-17545) was dissolved in dimethyl sulfoxide. Fibroblasts were incubated with LGK974 (1 μm) for 12 h. For in vivo assays, LGK974 was formulated in 0.5% MC/0.5% Tween 80 and administered by oral gavage at 3 mg/kg per day.

### Statistical analyses

Statistical significance was calculated with GraphPad Prism 6.0 (GraphPad Software, Inc., San Diego, CA, USA). Data are represented as the mean ± standard deviation, unless otherwise indicated. Experiments were analyzed using an unpaired Student’s *t* test for two groups. Where more than two groups were compared, one-way analysis of variance was used. A value of *P* < 0.05 was considered statistically significant.

## Electronic supplementary material

Supplementary Figure S1

Supplementary Figure S2

Supplementary Figure S3

Supplementary Figure S4

Supplementary Figure S5

Supplementary Figure S6

Supplementary Figure legends

Clinical history of human subjects

Primers used for quantitative PCR and RT-qPCR
