# Correction: Exosomal Wnt-induced dedifferentiation of colorectal cancer cells contributes to chemotherapy resistance

**DOI:** 10.1038/s41388-019-0863-x

**Published:** 2019-07-31

**Authors:** Y.-B. Hu, C. Yan, L. Mu, Y.–L. Mi, H. Zhao, H. Hu, X.-L. Li, D.-D. Tao, Y.-Q. Wu, J.-P. Gong, J.-C. Qin

**Affiliations:** 1 Department of Surgery, Tongji Hospital, Tongji Medical College, Huazhong University of Science and Technology, Wuhan, 430030 China; 2 Molecular Medicine Center, Tongji Hospital, Tongji Medical College, Huazhong University of Science and Technology, Wuhan, 430030 China

**Keywords:** Cancer stem cells, Cancer therapeutic resistance, Cell biology, Cancer microenvironment

**Correction to: Oncogene**

10.1038/s41388-018-0557-9 Published online 2 November 2018

Since the publication of this paper, the authors noticed that the original version of this article contains errors in Figs. 2b and 3b.

In Fig. 2b, the authors noted that the treatment in SW620 cells was listed mistakenly as CAF2-exo. The correct information is 18Co-exo.

**Fig. 2:**
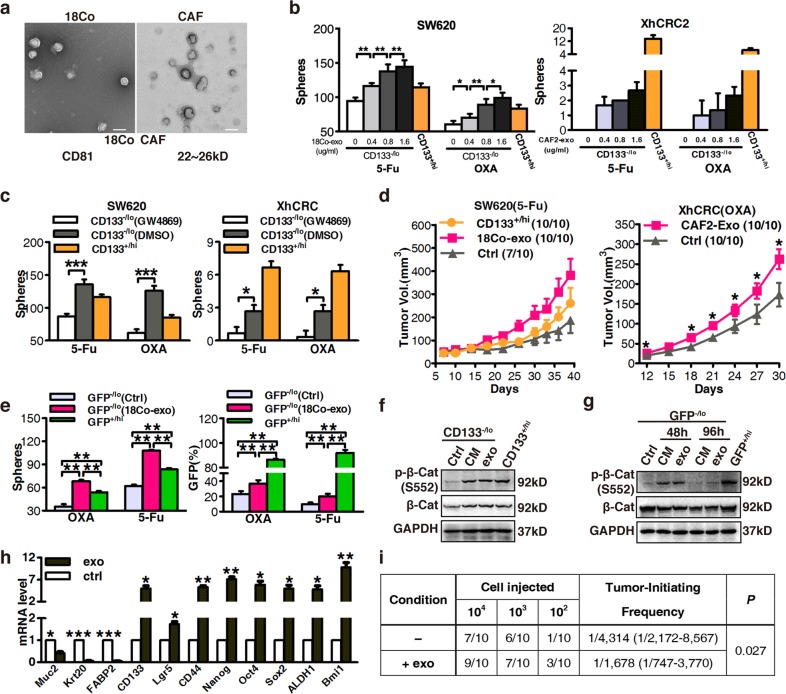
Exosomes contribute to the dedifferentiation of differentiated CRC cells and subsequent drug resistance. **a** Electron micrograph of exosomes isolated from 18Co cells and CAFs (top; scale bar, 100 nm) and immunoblotting analysis of the exosome marker CD81 (bottom). **b** The sphere-forming capacity of CD133−/lo SW620 or XhCRC2 cells treated with indicated concentrations of exosomes during chemotherapy (5-Fu or OXA), with CD133+/hi cells as the positive control. *P < 0.05, **P < 0.01. **c** CM derived from GW4869-pretreated fibroblasts was added to CD133−/lo CRC cells in a sphere formation assay, and CD133+/hi CRC cells were used as a positive control. *P < 0.05, ***P < 0.001. **d** Effects of exosomes on the growth of CD133−/lo CRC cells (1 × 105 SW620 cells or 4 × 105 XhCRC2 cells) inoculated into immunocompromised mice (n = 5) upon administration of 5-Fu or OXA. Tumor growth curves are shown, and CD133+/hi cells were used as a positive control in SW620 cells. *P < 0.05. **e** The sphere-forming capacity of GFP−/lo SW620 cells with or without 18Co-exosomes during chemotherapy (5-Fu or OXA), with GFP+/hi cells as a positive control. TOP-GFP expression of spheres was analyzed by flow cytometry. **P < 0.01. **f** Immunoblotting of total β-catenin and S552-phosphorylated β-catenin in CD133−/lo SW620 cells after stimulation with 18Co-CM or exosomes, and CD133+/hi CRC cells as positive control. **g** Immunoblotting of total β-catenin and S552-phosphorylated β-catenin in GFP−/lo SW620 cells after stimulation with 18Co-CM or exosome for 48 h and in the absence of CM or exosome for another 48 h. **h** mRNA levels of several differentiation markers (mucin2, cytokeratin 20, FABP2) and CSC makers (CD133, Lgr5, CD44, Nanog, OCT4, SOX2, ALDH1 and Bmi1) in exosome-treated spheres in CD133−/lo XhCRC cells. *P < 0.05. **P < 0.01, ***P < 0.001. **i** Tumor-initiating frequency of exosome-treated CD133−/lo XhCRC cells in NOD/SCID mice

In Fig. 3b, the colours of the labels representing the low risk and high risk groups did not match and the numbers of the patients were incorrect.

**Fig. 3:**
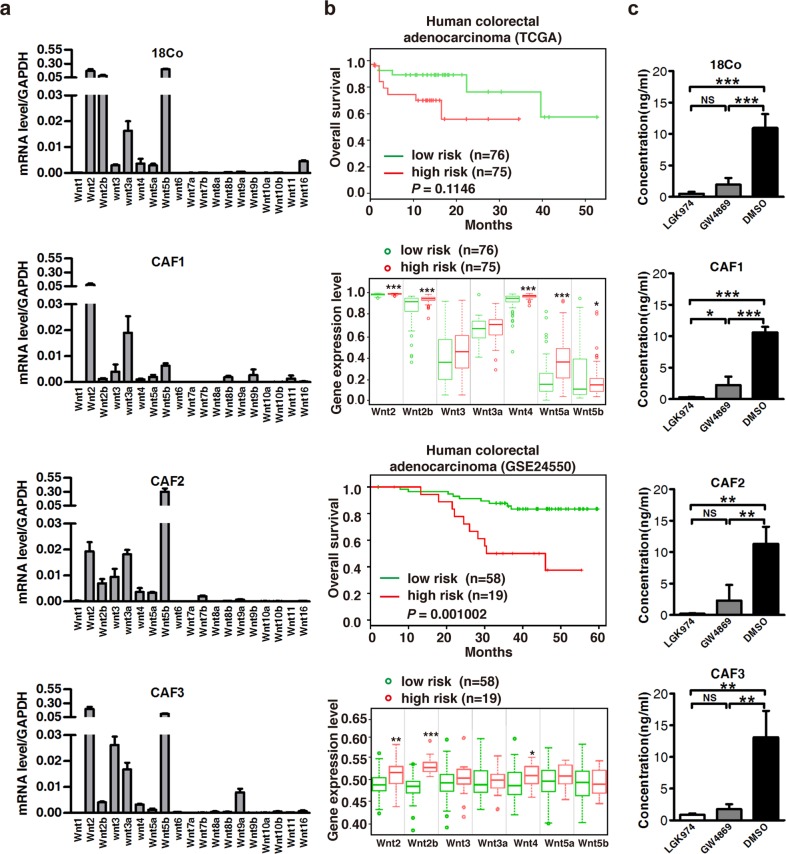
Wnts are secreted in fibroblast-exosomes and predict a poor prognosis. **a** Real-time PCR analysis of RNA from fibroblasts for Wnt mRNA expression. Wnt2, Wnt2b, Wnt3, Wnt3a, Wnt4, Wnt5a and Wnt5b were expressed in all four fibroblasts (18Co cells, CAF1, CAF2, and CAF3). **b** Kaplan–Meier curves showing that high expression of Wnt2, Wnt2b, Wnt3, Wnt3a, Wnt4, Wnt5a, and Wnt5b in two microarray data sets are positively associated with poor patient survival (P = 0.115 in TCGA and P = 0.001 in GSE24550). **c** Enzyme-linked immunosorbent assay (ELISA) to detect Wnt3a in exosomes from LGK974- or GW4869-treated 18Co cell-derived CM. **P < 0.01

The corrected figures are provided below.

The conclusions of this paper were not affected. The authors sincerely apologize for these errors and any confusion caused.

