# Supplementary figures and images for: Exosomal Wnt-induced dedifferentiation of colorectal cancer cells contributes to chemotherapy resistance
Source: Oncogene. 2018 Nov 2;38(11):1951–65. doi: 10.1038/s41388-018-0557-9 (PMC6756234; doi:10.1038/s41388-018-0557-9)

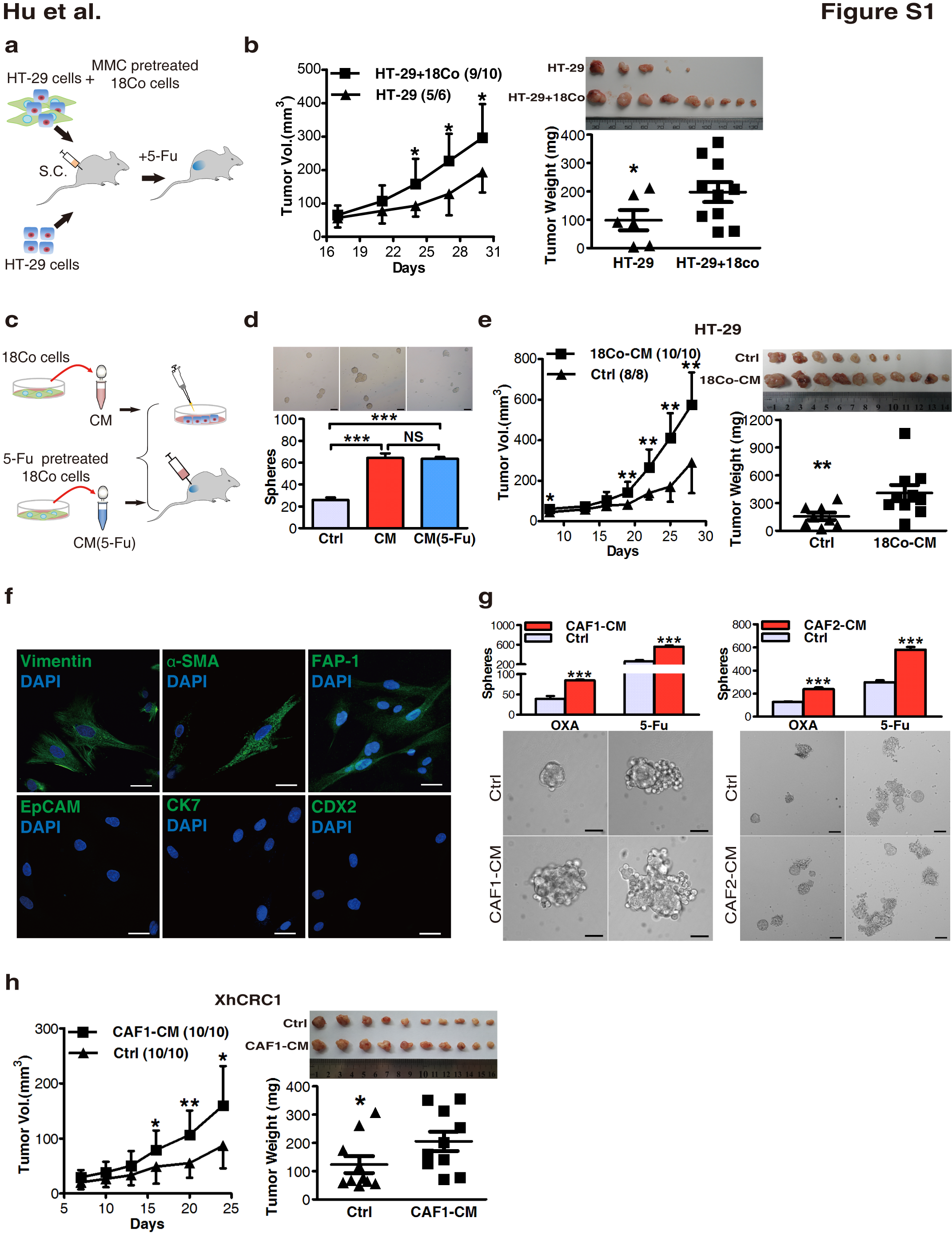

Supplement: Supplementary file 1 — Supplementary Figure S1 [file 41388_2018_557_MOESM1_ESM.tif]

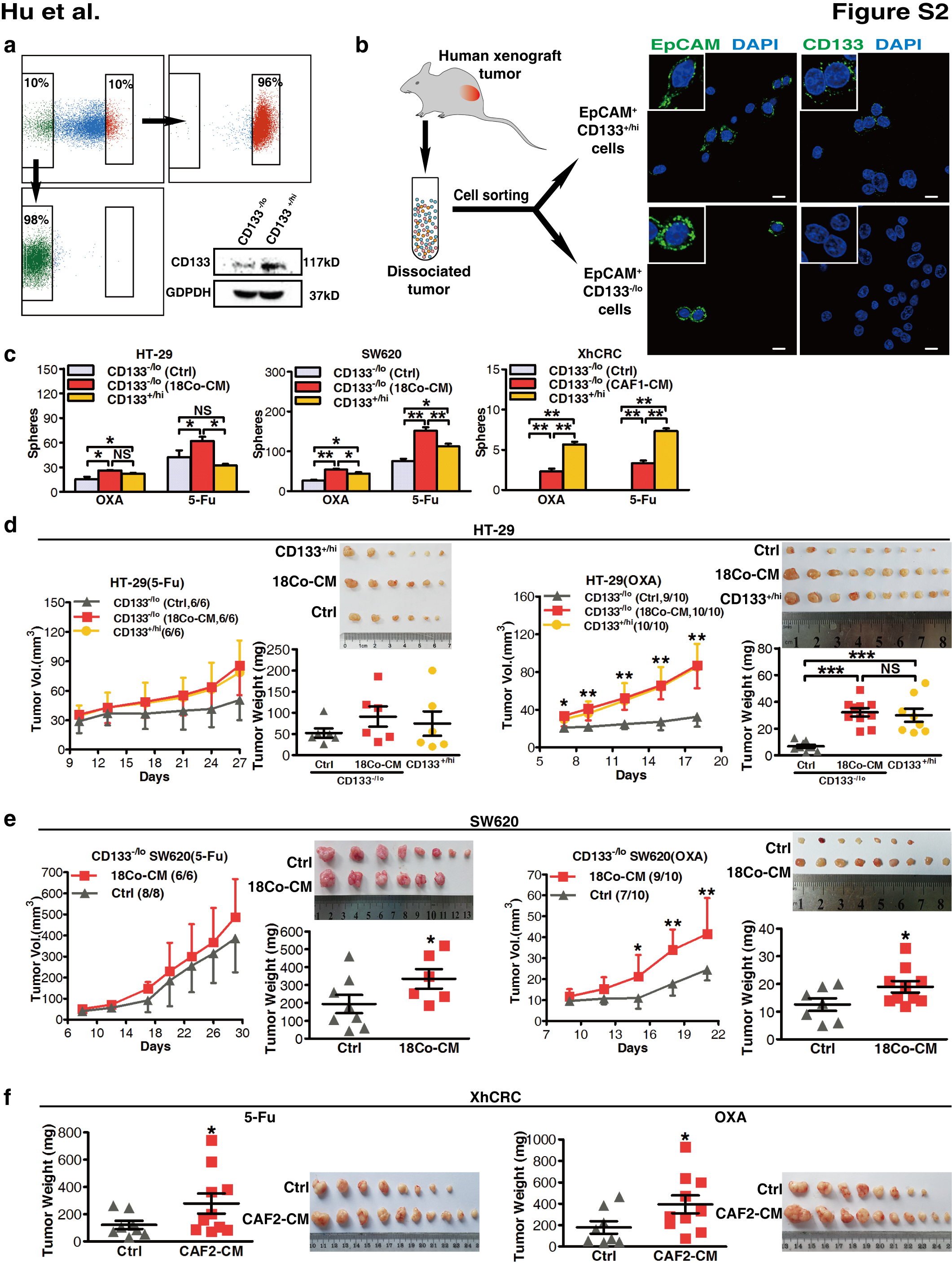

Supplement: Supplementary file 2 — Supplementary Figure S2 [file 41388_2018_557_MOESM2_ESM.tif]

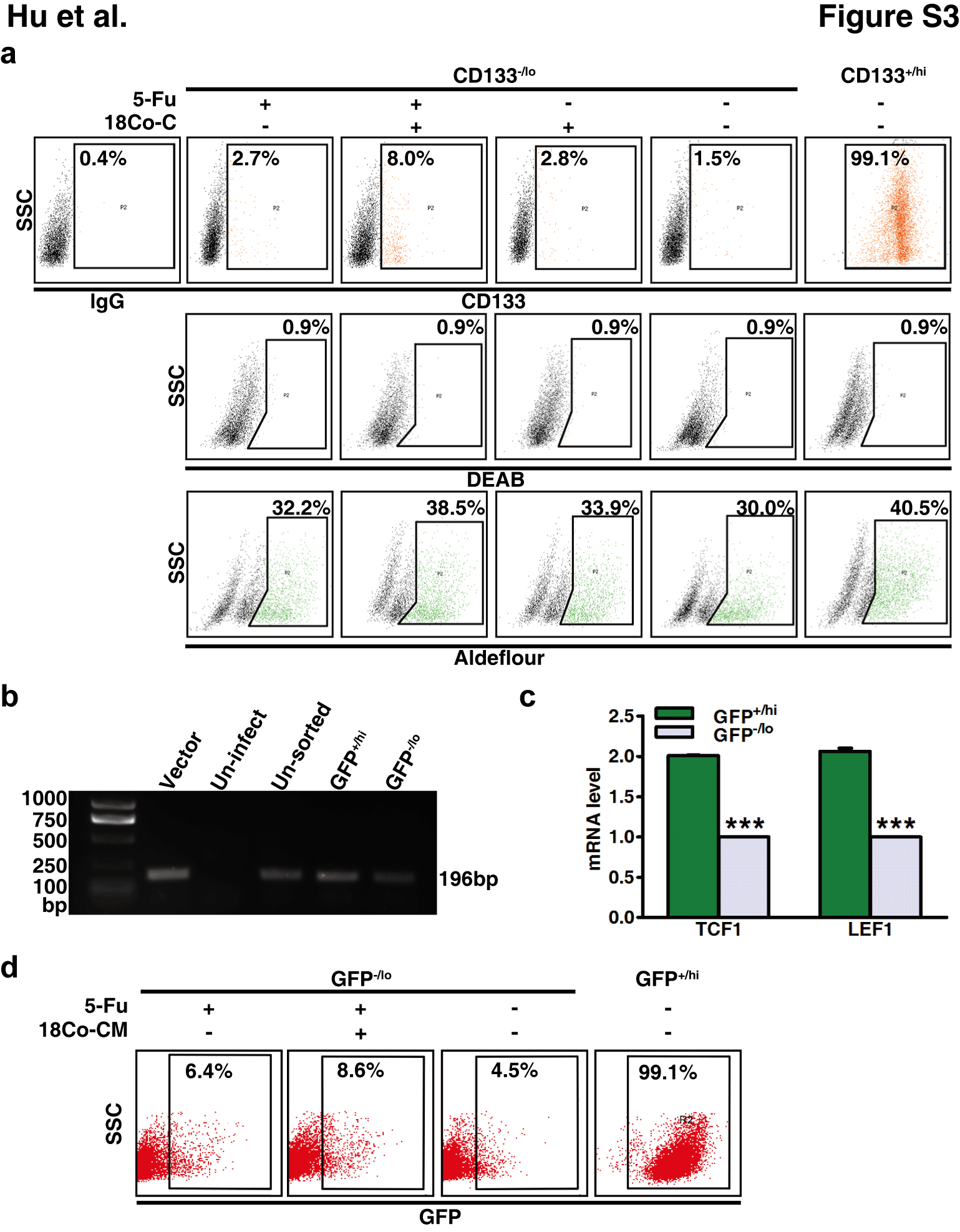

Supplement: Supplementary file 3 — Supplementary Figure S3 [file 41388_2018_557_MOESM3_ESM.tif]

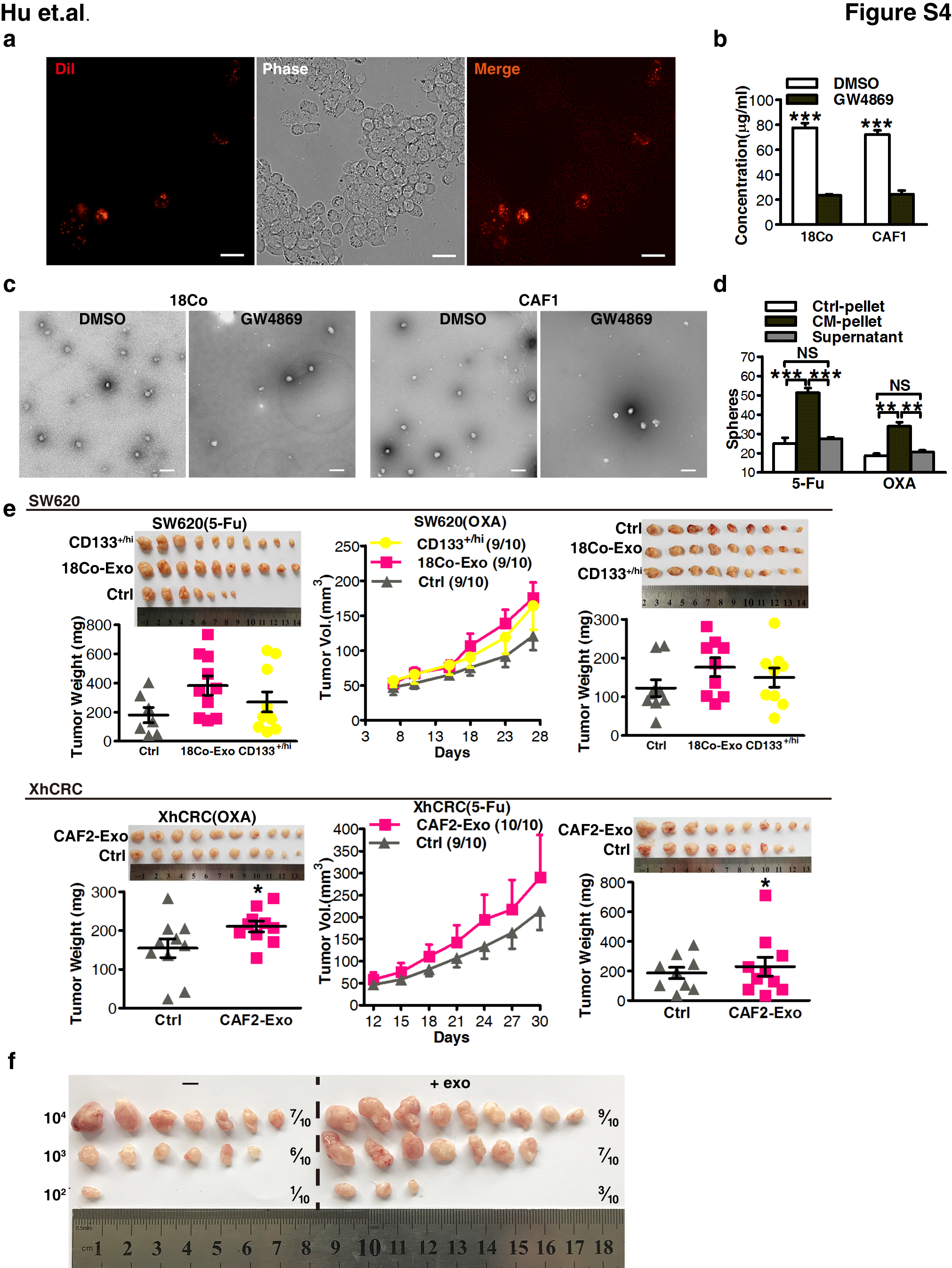

Supplement: Supplementary file 4 — Supplementary Figure S4 [file 41388_2018_557_MOESM4_ESM.tif]

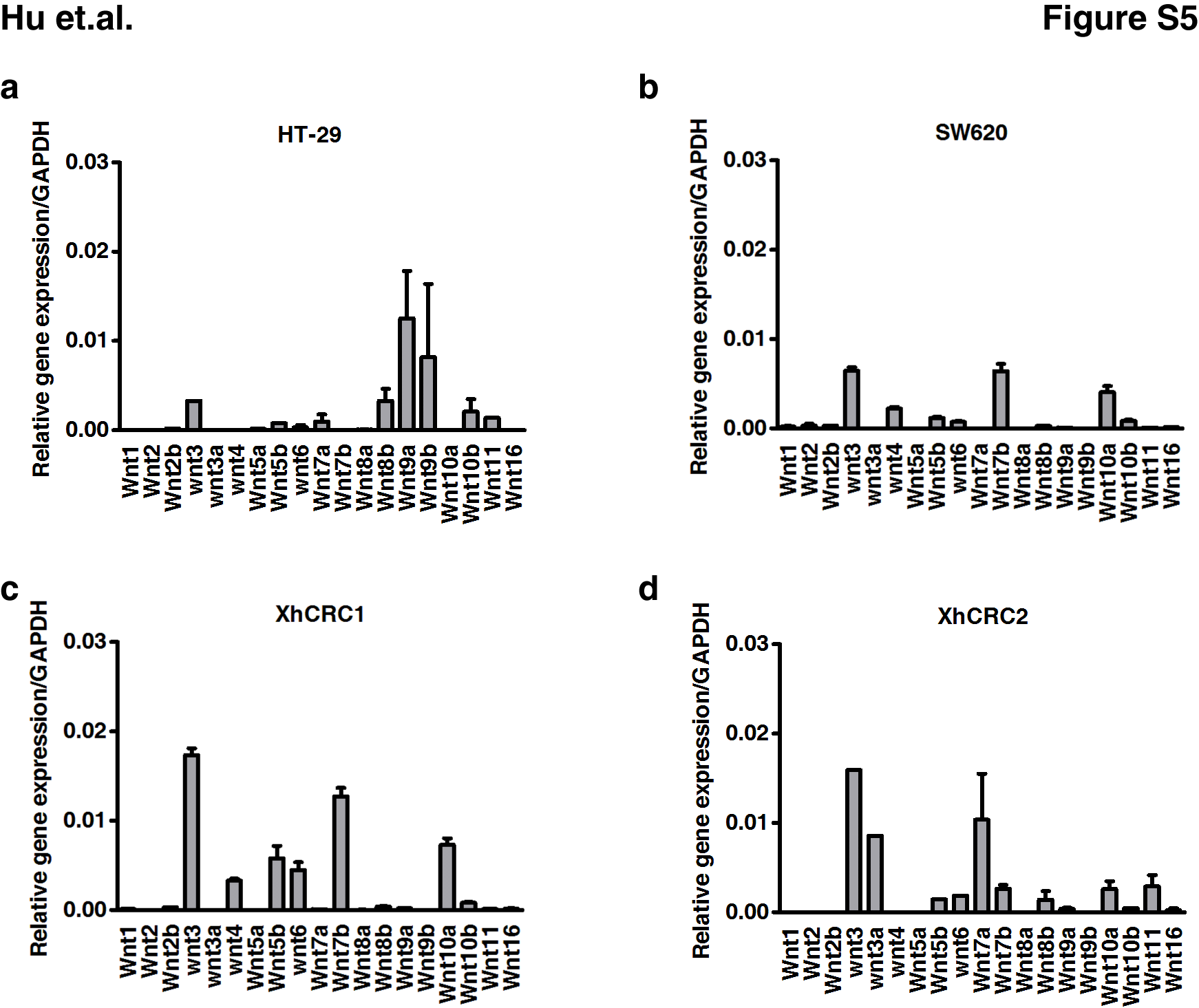

Supplement: Supplementary file 5 — Supplementary Figure S5 [file 41388_2018_557_MOESM5_ESM.tif]

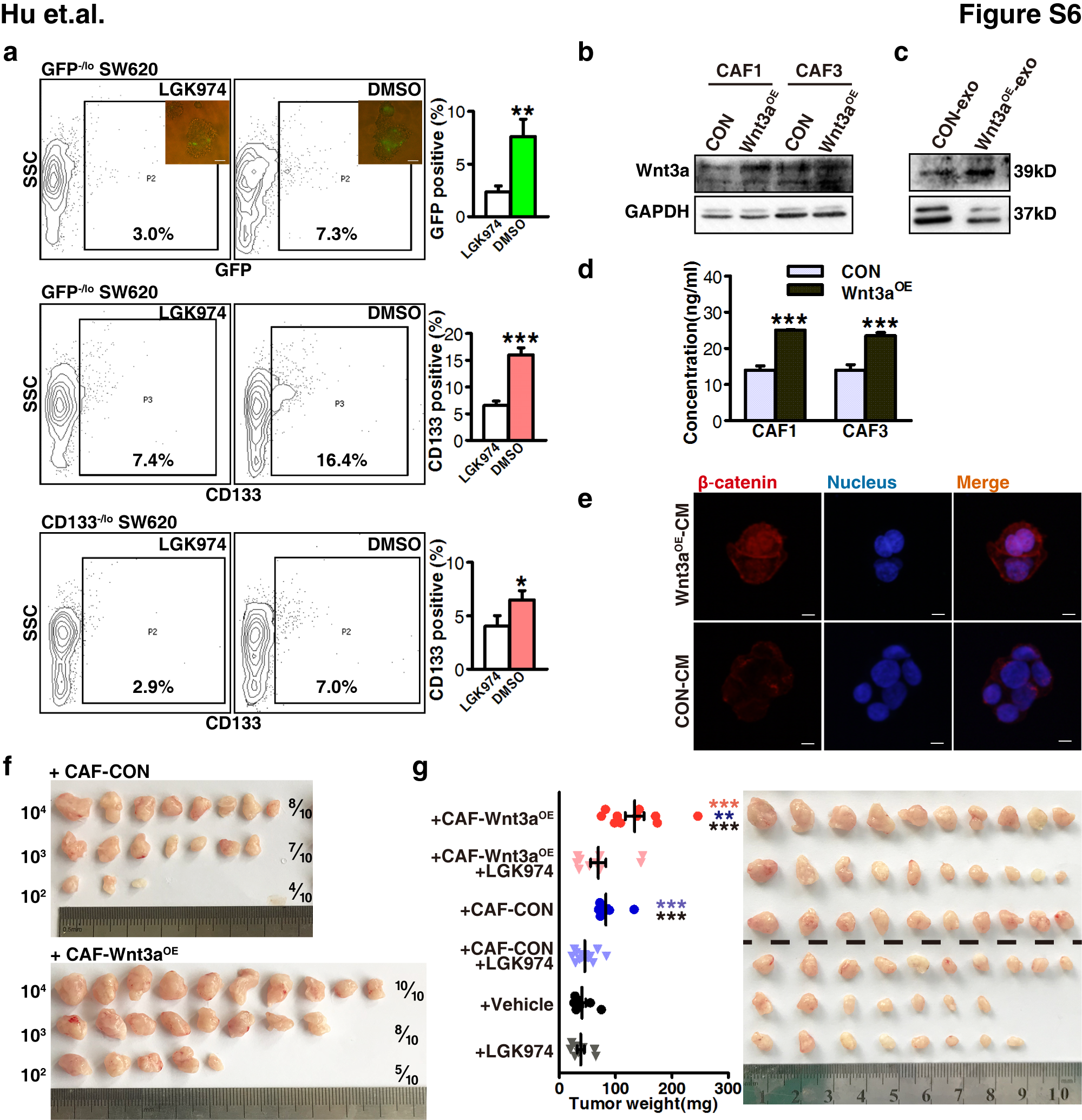

Supplement: Supplementary file 6 — Supplementary Figure S6 [file 41388_2018_557_MOESM6_ESM.tif]
