# Supplementary material for: Exosomal Wnt-induced dedifferentiation of colorectal cancer cells contributes to chemotherapy resistance
Source: Oncogene. 2018 Nov 2;38(11):1951–65. doi: 10.1038/s41388-018-0557-9 (PMC6756234; doi:10.1038/s41388-018-0557-9)
Supplement: Supplementary file 7 — Supplementary Figure legends [file 41388_2018_557_MOESM7_ESM.docx]

**SUPPLEMENTARY FIGURE LEGENDS**

**Figure S1** (related to Figure 1). **Fibroblasts promote drug resistance in CRC cells via paracrine signaling.**

**(a)** Experimental schematic: HT-29 cells (1×10^3^ cells) were injected into nude mice (three to five mice per group) with or without 18Co cells (1×10^3^ cells), which were pretreated with mitomycin C (MMC), and all mice were treated with 5-Fu.

**(b)** Tumor growth curves and tumor weights are shown. Representative tumors at the endpoint are inset in the right panel. **P* < 0.05.

**(c)** Experimental schematic: 18Co cells were pretreated with 5-Fu or control vehicle, and then CM was harvested from the two differently-treated 18Co cells. CM, conditioned medium harvested from 18Co cells cultured with fresh DMEM/F12. CM (5-Fu), conditioned medium harvested from 18Co cells pretreated with 5-Fu.

**(d)** The sphere-forming capacity of HT-29 cells treated with CM (5-Fu) or CM upon administration of 5-Fu. The inserts are representative microscopic images. Scale bar, 100 μm. ****P* < 0.001.

**(e)** Effects of 18Co-CM on the growth of HT-29 cells (1×10^3^ cells) inoculated into 5-Fu treated nude mice (four to five mice per group). Tumor growth curves and tumor weights are shown. Representative tumors at the endpoint are inset in the right panel. **P*< 0.05, ***P* < 0.01.

**(f)** Immunostaining of cancer associated fibroblasts (CAFs) cultured from primary EpCAM^-^ cells. Top: positive immunostaining for fibroblast makers (Vimentin, α-SMA and FAP-1); bottom: negative immunostaining for epithelial markers (EpCAM, CK7, and CDX2). Scale bars, 30 μm.

**(g)** The sphere-forming capacity of XhCRC cells treated with conditioned medium derived from primary CAFs upon administration of 5-Fu or OXA. Representative microscopic images are shown. Scale bar, 100 μm. ****P* < 0.001.

**(h)** Effects of CAF1-CM on the growth of XhCRC1 cells (1×10^6^ cells) inoculated into OXA-treated NOD/SCID mice (n = 5 per group). Tumor growth curves and tumor weights are shown. Representative tumors at the endpoint are inset in the right panel. **P*< 0.05, ***P* < 0.01.

**Figure S2** (related to Figure 1). **Fibroblasts promote drug resistance in differentiated CRC cells via paracrine signaling.**

**(a)** Representative FACS files for post-sort analysis of the sorted CD133^+/hi^ and CD133^-/lo^ CRC cells. Immunoblotting analysis of CD133 in purified CD133^+/hi^ and CD133^-/lo^ SW620 cells.

**(b)** Schematic of the sorting of CD133^+/hi^ and CD133^-/lo^ tumor cells from dissociated colorectal xenograft tumors by FACS. Immunostaining of purified CD133^+/hi^ and CD133^-/lo^ XhCRC cells for epithelial markers (EpCAM) and stem marker (CD133). Top: EpCAM^+^ CD133^+/hi^ XhCRC cells; bottom: EpCAM^+^ CD133^-/lo^ XhCRC cells. Scale bars, 10 μm.

**(c)** Effects of fibroblast-derived conditioned medium (CM) on the sphere-forming capacity of CD133^-/lo^ CRC cells during chemotherapy (OXA or 5-Fu), and CD133^+/hi^ cells were used as a positive control. **P* < 0.05, ***P* < 0 .01.

**(d–f)** CD133^-/lo^ CRC cells (1×10^4^ HT-29 cells, 5×10^4^ SW620 cells, or 5×10^5^ XhCRC1 cells) were implanted into immunocompromised mice (three to five mice per group) upon administration of 5-Fu or OXA. Mice were then treated with or without CM and CD133^+/hi^ cells were used as a positive control in HT-29 cells. Tumor growth curves, tumors and their volumes are shown. **P* < 0.05, ***P*< 0.01, ****P* < 0.001.

**Figure S3** (related to Figure 1)**. Fibroblast-secreted factors endow differentiated CRC cells with stem cell-like properties.**

**(a)** CD133^-/lo^ SW620 cells treated with 18Co-CM *in vitro* were analyzed by flow cytometry to determine the expression of the stem cell maker CD133 and the functional stem cell factor Aldeﬂuor (ALDH).

**(b)** Genomic PCR of *GFP* sequence in GFP^+/hi^ and GFP^-/lo^ SW620 cells; unsorted cells were used as a positive control for GFP.

**(c)** Quantitative real-time PCR analysis of *TCF* and *LEF* mRNA in GFP^+/hi^ and GFP^-/lo^ SW620 cells. ****P* < 0.001.

**(d)** GFP^-/lo^ SW620 cells were cultured with or without 18Co-CM upon administration of 5-Fu, or cultured with 18Co-CM without chemotherapy. And GFP^+/hi^ cells as positive control. GFP expression was analyzed by flow cytometry.

**Figure S4** (related to Figure 2)**. Exosomes contribute to the dedifferentiation of differentiated CRC cells and subsequent drug resistance.**

**(a)** DiI-labeled exosomes derived from 18Co cells were added to SW620 cells. After incubation for 24 h, images were obtained. Scale bar, 30 μm.

**(b)** Concentration of exosomes in CM from GW4869- or DMSO-treated 18Co cells and CAF1. ****P* < 0.001.

**(c)** Electron micrograph of exosomes isolated from GW4869- or DMSO-pretreated 18Co cells and CAF1. (scale bar, 500 nm).

**(d)** The sphere-forming capacity of CD133^-/lo^ SW620 cells treated with 18Co-derived exosomes by ultracentrifugation or exosome-depleted supernatants during chemotherapy (5-Fu or OXA). ***P* < 0.01, ****P* < 0.001.

**(e)** CD133^-/lo^ CRC cells (1×10^5^ SW620 cells or 4×10^5^ XhCRC2 cells) were subcutaneously implanted into immunocompromised mice (n = 5 per group) upon administration with OXA or 5-Fu. Mice were then treated with or without exosomes isolated from fibroblasts (18Co cells in SW620 xenograft and CAF2 in XhCRC xenograft), and CD133^+/hi^ cells were used as a positive control in SW620 cells. Tumor weight and growth curves are shown. **P* < 0.05.

**(f)** Tumor-initiating frequency of exosome-treated CD133^-/lo^ XhCRC cells in NOD/SCID mice.

**Figure S5** (related to Figure 3)**. Real-time PCR analysis of Wnts mRNA level in four CRC cells.**

**(a)** HT-29 cells; **(b)** SW620 cells; **(c)** XhCRC1 cells; **(d)** XhCRC2 cells.

**Figure S6** (related to Figure 4)**. Exosomal Wnts protect differentiated CRC cells against chemotherapy.**

**(a)** Exosomes were derived from 18Co cells pretreated with LGK974 and then added to differentiated SW620 cells (CD133^-/lo^ or GFP^-/lo^ cells) treated with OXA. Top: The percentages of GFP-positive cells in GFP^-/lo^ SW620 spheres were analyzed by flow cytometry. Representative microscopic images of Wnt activity are inset. Scale bars, 50 μm. Middle: The percentages of CD133-positive cells in GFP^-/lo^ SW620 spheres were analyzed by flow cytometry. Bottom: The percentages of CD133-positive cells in CD133^-/lo^ SW620 spheres were analyzed by flow cytometry. **P* < 0.05, ***P* < 0.01, ****P* < 0.001.

**(b)** Immunoblotting of Wnt3a in CAFs transfected with Wnt3a-overexpressing lentivirus or control lentivirus.

**(c)** Immunoblotting of Wnt3a in exosomes derived from CAF3 transfected with Wnt3a-overexpressing lentivirus or control lentivirus.

**(d)** ELISA to detect Wnt3a in exosomes derived from Wnt3a overexpressed CAFs. ****P* < 0.001.

**(e)** Wnt3a-overexpressing CAF3-derived CM was added to differentiated XhCRC cells (CD133^-/lo^ XhCRC2 cells) upon treatment with OXA. The nuclear translocation of β-catenin in differentiated XhCRC cells treated with Wnt3a^OE^-CM was estimated. Representative images of spheres stained with β-catenin (red). Nuclei were stained with DAPI (blue). Scale bars, 10 µm.

**(f)** Tumor-initiating frequency of CD133^-/lo^ XhCRC cells co-implanted with Wnt3a-overexpressed CAF3 in NOD/SCID mice.

**(g)** CD133^-/lo^ XhCRC2 cells (6×10^4^ cells) were injected into NOD/SCID mice (n = 5 per group) with or without 6×10^4^ Wnt3a^OE^-CAF3 or control CAF3, and all mice were intraperitoneally treated with OXA and orally fed with LGK974 or control vehicle. Tumors and tumor weights are shown. Data are presented as mean ± SEM.**P* < 0.05, ***P* < 0.01.
