# Supplementary material for: Exosomal Wnt-induced dedifferentiation of colorectal cancer cells contributes to chemotherapy resistance
Source: Oncogene. 2018 Nov 2;38(11):1951–65. doi: 10.1038/s41388-018-0557-9 (PMC6756234; doi:10.1038/s41388-018-0557-9)
Supplement: Supplementary file 8 — Clinical history of human subjects [file 41388_2018_557_MOESM8_ESM.docx]

Table S1. Clinical history of human subjects

| **Patient**  **No.** | **Age** | **Gender** | **TNM** | **Tumor**  **Grading** |
| --- | --- | --- | --- | --- |
| **CAF1** | 54 | Female | T4N0(0/14)M0 | G2 |
| **CAF2** | 53 | Male | T4N1a(1/13)M0 | G2-G3 |
| **CAF3** | 69 | Female | T2bN0(0/14)M0 | G2 |
| **XhCRC1** | 47 | Female | T4bN2(4/10)M0 | G2 |
| **XhCRC2** | 43 | Female | T4aN0(0/14)M0 | G2-G3 |
