# Supplementary material for: Exosomal Wnt-induced dedifferentiation of colorectal cancer cells contributes to chemotherapy resistance
Source: Oncogene. 2018 Nov 2;38(11):1951–65. doi: 10.1038/s41388-018-0557-9 (PMC6756234; doi:10.1038/s41388-018-0557-9)
Supplement: Supplementary file 9 — Primers used for quantitative PCR and RT-qPCR [file 41388_2018_557_MOESM9_ESM.docx]

Table S2. Primers used for quantitative PCR and RT-qPCR.

| **PCR** | | | |
| --- | --- | --- | --- |
| **Genes** | | **5`Primer** | **3`Primer** |
| EGFP | | GAAGAACGGCATCAAGGTG | CTCCAGCAGGACCATGTGA |
| GAPDH | | TCGTGGAAGGACTCATGACC | TCCACCACCCTGTTGCTGTA |
|  | | | |
| **RT-qPCR** | | | |
| **Genes** | **5`Primer** | | **3`Primer** |
| CD133 | GCCACCGCTCTAGATACTGC | | TGTTGTGATGGGCTTGTCAT |
| CD44 | AGCAACCAAGAGGCAAGAAA | | GTGTGGTTGAAATGGTGCTG |
| Bmi1 | AAATGCTGGAGAACTGGAAAG | | CTGTGGATGAGGAGACTGC |
| Sox2 | TACAGCATGTCCTACTCGCAG | | GAGGAAGAGGTAACCACAGGG |
| ALDH1 | GCACGCCAGACTTACCTGTC | | CCTCCTCAGTTGCAGGATTAAAG |
| Nanog | CAACCAGACCCAGAACATCC | | TTCCAAAGCAGCCTCCAAG |
| OCT4 | ACCGAGTGAGAGGCAACC | | TGAGAAAGGAGACCCAGCAG |
| TCF1 | CGGGACAGAGGACCATTACAACTAGATCAAGGAG | | CCACCTGCCTCGGCCTGCCAAAGT |
| LEF1 | AATGTAATAGCCAAACCCACTCT | | AACGCATCTGTTAAGGTTTACTGT |
| Lgr5 | CTGCCTGCAATCTACAAGGT | | CCCTTGGGAATGTATGTCAGA |
| Survivin | GCCCAGTGTTTCTTCTGCTT | | CCGGACGAATGCTTTTTATG |
| Axin | CTCCTTATCGTGTGGGCAGT | | CTTCATCCTCTCGGATCTGC |
| Mucin2 | CGAAACCACGGCCACAACGT | | GACCACGGCCCCGTTAAGCA |
| Krt20 | TGTCCTGCAAATTGATAATGCT | | AGACGTATTCCTCTCTCACTCTCATA |
| FABP2 | TGGAAGGTAGACCGGAGT | | AGGTCCCCCTGAGTTCAGTT |
| c-Met | CTGCCTGCAATCTACAAGGT | | ATGGTCAGCCTTGTCCCTC |
| Wnt1 | CGGCGTTTATCTTCGCTATC | | CACGTGCAGGATTCGATG |
| Wnt2 | GCCACACGCTGCACCTAAAGC | | CAATTACCCTAAGGGTGGTAGC |
| Wnt2b | CGGGACCACACCGTCTTTG | | GCGAGTAATAGCGTGGACTAC |
| Wnt3 | AGGGCACCTCCACCATTTG | | GACACTAACACGCCGAAGTCA |
| Wnt3a | AGCTACCCGATCTGGTGGTC | | CAAACTCGATGTCCTCGCTAC |
| Wnt4 | AGGAGGAGACGTGCGAGAAA | | CGAGTCCATGACTTCCAGGT |
| Wnt5a | AGGGCTCCTACGAGAGTGCT | | TCACCGCGTATGTGAAGGC |
| Wnt5b | CATGGCCTACATAGGGGAGG | | CTGTGCTGCAATTCCACCG |
| Wnt6 | GGTGCGAGAGTGCCAGTTC | | CGTCTCCCGAATGTCCTGTT |
| Wnt7a | CTGTGGCTGCGACAAAGAGAA | | GCCGTGGCACTTACATTCC |
| Wnt7b | GAAGCAGGGCTACTACAACCA | | CGGCCTCATTGTTATGCAGGT |
| Wnt8a | GAACCTGTTTATGCTCTGGGC | | CAGCGTTCCCAAGCAAACTG |
| Wnt8b | CCGACACCTTTCGCTCCATC | | CAGCCCTAGCGTTTTGTTCTC |
| Wnt9a | GGCAAGCATCTGAAGCACAAG | | GCAGAAGCTAGGCGAGTCA |
| Wnt9b | TGTGCGGTGACAACCTCAAG | | ACAGGAGCCTGATACGCCAT |
| Wnt10a | AGATCGCCATCCACGAATGC | | ATCTTGTTGCGAGTCTCCAGG |
| Wnt10b | GTGAGCGAGACCCCACTATG | | CACTCTGTAACCTTGCACTCATC |
| Wnt11 | GGAGTCGGCCTTCGTGTATG | | GCCCGTAGCTGAGGTTGTC |
| Wnt16 | TTCAGACACGAGAGATGGAACT | | CCAGCCTTCACTTGCTGAG |
| GAPDH | TCGTGGAAGGACTCATGACC | | TCCACCACCCTGTTGCTGTA |
